# Low cost 3D printing of metals using filled polymer pellets

**DOI:** 10.1016/j.ohx.2022.e00292

**Published:** 2022-03-12

**Authors:** Vincent Martin, Jean-François Witz, Frédéric Gillon, Denis Najjar, Philippe Quaegebeur, Abdelkader Benabou, Michel Hecquet, Emmanuel Berté, François Lesaffre, Matthieu Meersdam, Delphine Auzene

**Affiliations:** a Univ. Lille, Arts et Metiers Institute of Technology, Centrale Lille, Junia, ULR 2697 – L2EP Lille, France; b Univ. Lille, CNRS, Centrale Lille, UMR 9013 – LaMcube – Laboratoire de Mécanique, Multiphysique, Multiéchelle, Lille, France; c CRITT-MDTS, Charleville-Mézières, France

**Keywords:** Low cost Metal 3D printing, Pellets additive manufacturing

## Abstract

Nowadays, additive manufacturing of metallic materials is most often carried out using expensive and complex tools that leave the user with limited control and no possibility of modification. In order to make the printing of metal parts more accessible to small structures but also better suited for academic research, the use of a mixture of thermoplastic polymer and metal powder is a good solution as many granular feedstocks already exist for Metal Injection Molding applications. To perform the shaping process, the Fused Granular Fabrication 3D printing technology is set up by diverting the use of a feedstock in the form of pellets that are directly inserted into the print head. This solution, which is less costly, is implemented here by modifying a mid-range printer, the Tool Changer from E3D, and by making the hardware and software adaptations to mount a compact granulates extruder on it, which is also available on the market. The polymer portion present in the green part can then be removed in order to perform the heat treatments that will densify the powder by sintering and give a fully metallic dense object.

## Graphical abstract

**Figure:**
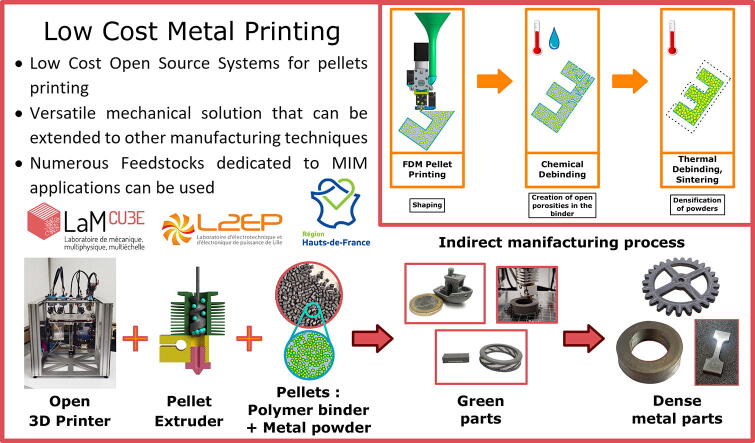

**Specifications table:** 

Hardware name	*Pellet metal 3D printer*
Subject area	• Engineering and materials science Educational tools and open source alternatives to existing infrastructure
Hardware type	• Mechanical engineering and materials science Electrical engineering Additive manufacturing
Closest commercial analog	*Pollen Additive Manufacturing MC printer; Direct 3D printer*
Open source license	Creative Commons Attribution – ShareAlike license.
Cost of hardware	*∼2600 €*
Source file repository	https://doi.org/10.5281/zenodo.5484803

## Hardware in context

The possibilities offered by additive manufacturing are now benefiting many industries that can take advantage of greater freedom of shape and standardized tools. One example is the creation of complete components with a single printer using multi-material printing technologies [1], [2], or the manufacture of lighter parts with complex geometries for the aerospace [3], [4] and automotive industries [5], [6]. These sectors can also benefit from a significant reduction in costs as well as greater flexibility in their production chain, particularly thanks to reduced set-up times and a more sparing use of matter [7], [8], [9]. Therefore, the application of 3D printing processes to the materials typically used in these fields is a crucial development point that has been the subject of much attention for several decades [10], especially for metallic [11], [12] and ceramic [13] materials. However, the shaping of metals by additive manufacturing processes remains a highly specialized activity whose equipment and implementation costs remain very high [14], [15]. Three processes that use a powder bed are frequently employed: Selective Laser Sintering (SLS) [16], Selective Laser Melting (SLM) [17] and Binder Jetting [18]. For each of them, the various stages of shaping require a significant investment in equipment. Commonly found prices for laser application range between two hundred and eight hundred thousand euros. In addition, the implementation of a feedstock in the form of a powder bed can be difficult to handle and requires strong control of the printing environment.

In contrast, Material Extrusion is an affordable and widely explored method. Some 3D printing solutions for metallic materials based on a loaded polymer feedstock are already commercially available. We can give the example of the MarkForged and Desktop Metal printing systems [19], which offer to shape polymers filled with metallic or ceramic powders using a plunger. Developments of similar systems can be found in the scientific literature as well. Volpato et. al. [20] developed and studied the behavior of a piston driven syringe used for the extrusion of polypropylene granules and Wijnen et al. [21] proposed an open source motorized syringe pump that is suitable for 3d printing applications [22], [23]. This system has been investigated as well by Li et. al. [24] to extrude loaded aqueous pastes in order to additively manufacture ceramic materials. The authors underlined the difficulties encountered to obtain precise start and stop positions of the extruded lines due to the large amount of matter to displace with the plunger. Similarly, it is reported that the presence of inhomogeneous area in the feedstock made it challenging to extrude a constant width line.

Other examples also employ polymer filaments filled with metal or ceramic powders that are intended to be printed using a conventional Fused Filament Fabrication (FFF) printer. We can name the Ultrafuse 316L produced by BASF [25], or the alumina filament produced by Zetamix^1^. However, few materials are currently available for this process, mainly because of the difficulties encountered when creating a powder highly filled filament [26] and when handling it. Indeed, the presence of grains of matter coming from a different nature than that of the polymer binder greatly weakens the material, especially for high filling rates. Furthermore, the development cost of a new filament filled with a different powder material can vary from five thousand to several tens of thousands of euros.

One solution to overcome the above-mentioned difficulties is to employ a process close to FFF, but for which the feedstock is used in the form of pellets. In this way, the brittleness of the composite material (polymer binder + metal powder) is no longer a problem. In addition, this allows one to benefit from the tools and experience of the large community that has been using 3D printing for almost 20 years. Several machines that employ this technique are commercially available. The company Pollen^2^ offers for sale a range of printers that use this process. Called PAM (Pellet Additive Manufacturing), it features a wide selection of composite polymer materials and his cost starts at 65000$^3^. Other solutions, more oriented towards industrial applications, exist as well such as the ATLAS printers from Titan robotics^4^, around 250000$. We can also mention the BAAM [27] system developed by Cincinnati Incorporated and the Oak Ridge National Laboratories as well as the robotic arm-mounted extrusion solutions provided by the company Weber^5^, which are intended for Large Format Additive Manufacturing. Indeed, the use of granules facilitates the storage and supply of the feedstock while allowing for higher flow rate [28]. This allows the implementation of larger building volumes as also illustrated by the Delta WASP 3MT^6^ printer that comes of a price of 49000$. Other desktop models can be found for lower budgets such as the BigFoot 200 from Tumaker^7^ for 6000$. Another example is the company Direct3D^8^, which produces a print head for extruding materials from granules. Finally, it is possible to acquire only an extruder and to adapt it on a three-axis cinematic system. The company Dyze Design^9^ sells the pulsar extruder for a little above 10,000$. This one features an important material extrusion flow and the temperature along its screw can be tuned through three heat zones. However, these machines remain expensive and the associated investment costs represent a major restriction for a private individual or a small structure that would like to acquire such an equipment. Their operation is strongly linked to the manufacturer's brand as they often have important software and hardware restrictions that do not allow the user much autonomy. Consequently, these processes are not well adapted to development work or proper academic research, which often require modifications of the equipment or the control chain. However, simpler and more affordable solutions, that only provides the pellet extruder, exist as well. We can give the example of the Bravo Basic from Polylab^10^, a printing head that retails for 2400$, or of the Lily Kit from Recycl3dprint^11^ which propose to assemble a similar extrusion system for about a thousand dollars. Finally, the Mahor pellet extruder, is available for about 500€. This last one is the solution used in this work to set up the Material Extrusion process.

In addition to that, such systems have been extensively studied in the literature. Different research works have implemented their own version of a pellet extruder for 3d printing, also known as Fused Granular Fabrication (FGF) or Fused Pellet Fabrication (FPF). A multi feed head was built by Zhou et. al. [29] for printing heat-sensitive materials, which allows to reduce the thermal degradation suffered by the polymer during various heat treatment. Similar developments have been made to print different kinds of polymers using custom extruder [30], [31]. Different points of interest are investigated such as the physical behavior of the materials (often necessary to obtain the correct printing parameters), the mechanical performances of the printed structures or the mechanisms at play in the cohesion between lines and between layers. In the same way, other works present the modeling of the physical phenomena that govern the conveying and the melting of the granules [32] as well as the extrusion quality of the material [33], [34]. Such points are essential for the fine tuning of the process and the development of this technology. Another application of the Material Extrusion Additive Manufacturing is to re-use plastic waste as a feedstock. Several works have been carried out on this theme and various aspects were investigated, such as the feasibility with respect to the particle size and the type of polymer, the mechanical performances or the economic impact of the process on the price of the parts [35], [36], [37], [38]. The machine used in these works was a Gigabot X,^12^ a mid-range open-source 3D printer that can print from a granular feedstock thanks to a miniaturize thermoplastic extruder head that also features three distinct heat zones.

As mentioned earlier, another important application of printing from granules is the extrusion and shaping of polymer filled with a powder of a different material. Firstly, this allows to introduce an additional functionality to the parts. Various examples can be found in the literature such as the work of Huber et. al. [39] or Li et. al. [40], [41] that respectively use loaded filament and pellets to additively build polymer loaded with neodymium particles. This allows them to obtain magnets with complex shapes that feature a specific magnetic field distribution. Finally, this type of feedstock can be used to produce dense metal parts through a process called MIM-like Additive Manufacturing. For this, the printed part will undergo heat treatments in order to remove the polymer binder and solidify the metal particles. These steps are similar to the ones used in Metal Injection Molding technologies (MIM, or more generally PIM for Powder Injection Molding [42]). Several research work have focused on this technique to produce parts made out of different type of metal such as AISI 630 [43] or 17-4 PH [44] stainless steel. 316L stainless steel parts have also been built using this process while the printing step was performed using a filament feedstock [45], [46] or granulates extruded using a motor-driven piston system [47]. Recently, Hassan et. al. [48] investigated the impact of the printing parameters and orientation on the mechanical properties, the micro-structure and the porosity level of the sintered part. Other materials are printed using Material Extrusion with the examples of ceramics like alumina [19], [49] or zirconia [43]. To go further, a large variety of materials can be used since this technique allows to divert feedstocks in the form of pellets originally intended for PIM applications. Indeed, a lot of the polymer binder used are thermoplastic with low fusion temperature. This provides a simpler way to obtain composite feedstock and partly eliminates the issue of development, giving access to a wide range of materials [19], [50], [51] that can be additively manufactured and densify thanks to a heat treatment.

Considering the current state of the art, it seems relevant to propose an affordable and efficient granular printing solution to support future developments, especially with regard to PIM-like metallic printing. In this work, the implementation of a low-cost solution for metal additive manufacturing using Fused Granular Fabrication from a feedstock in the form of pellets is presented. All the necessary components, the adaptations parts as well as the parameters setting and the control of the whole are detailed. In order to illustrate the functioning of the proposed printing system, green parts are produced using 316L Stainless Steel (SS) polymer granulates sold by the PolyMIM company.

The choice of the mechanical base and software system have been made in order to follow the Open Source Hardware (OSH) approach. This OSH initiative, based on modular and easily reproducible systems components, is strongly linked with early additive manufacturing technologies and still drives a large part of the projects that are linked to it. Numerous examples can be found inside the Maker community and the RepRap project [52]. Technical solutions designed with these principles are particularly useful for academic research activities [53] and for any structure that needs advanced versatile systems that can be modified extensively [54], [55], [56]. Good examples of open source designs applied to metal 3D printing can be found in the recent works done on Wire Arc Additive Manufacturing (WAAM) [57], [58], [59]. Here, Gas Metal Arc Welding system are associated with 3D CNC system in order to build part by directly depositing fused metal lines.

In our case, the part details and drawings are given by the manufacturer as well as the electronic designs. The chosen components are both inexpensive (especially if compared with other 3d printing control board or other multi tool systems) and well-made. The rest of the design presented here tries to give every useful bit of information going from CAD parts and configuration code to commissioning and usage advice. The objective of this article is to give a way and to facilitate the implementation of the shaping of loaded pellets for an investment lower than three thousand euros. It is aimed at committed enthusiasts, the scientific community and small companies. The installation and adjustment of the machine nevertheless requires a significant amount of development time. The purpose of this guide is to help the user to go faster in the assembly and adjustment steps in order to obtain a functional machine in a set-up time that we estimate to be around 200 h.

### Main principle

FFF is one of the first additive manufacturing processes developed [60]. It works by inserting solid feedstock into a moving print head, which melts the material and deposits it as a viscous liquid. The movement of the head, synchronized with the flow of extruded material, allows to build the geometry of the part. For this purpose, the object is broken down into several horizontal layers that are successively created. Each layer is formed by a complex pattern along which the material filaments are extruded. The filaments, which are still hot, bond with each other and with the already printed layer during the application process, thus ensuring the cohesion of the part. The material can also be cooled as soon as it leaves the nozzle to solidify it faster and freeze the resulting shape.

The conventional method of feeding the material to the extrusion head is to use a solid filament stored as a spool. This way, the polymer wire is driven by pinching it between a roller and a gear wheel, whose position is controlled by a motor. However, the proper functioning of this process relies heavily on the quality of the filament. Its diameter must be known and vary as little as possible to ensure a good control of the flow. Most 1.75 mm diameter wires have a tolerance of 50 µm. The filament must also be easy to handle in order to be fed to the extrusion body and is required to be flexible enough in order to wind it. The most common materials on the market are polymers with a relatively low melting point (between 200 and 300 °C), which can be prepared as a filament spool due to their elastic behavior.

To implement 3D printing by Material Extrusion of filled materials using, the addition of metallic or ceramic particles in a polymer matrix is carried out but, as mentioned above, this mixture can hardly be used as a filament. In order to be able to use this formulation (polymers and metal powders) in the form of small pellets, an adaptation of the extrusion head is necessary. Especially for the heating and pellet feed elements.

**Fig. 1:**
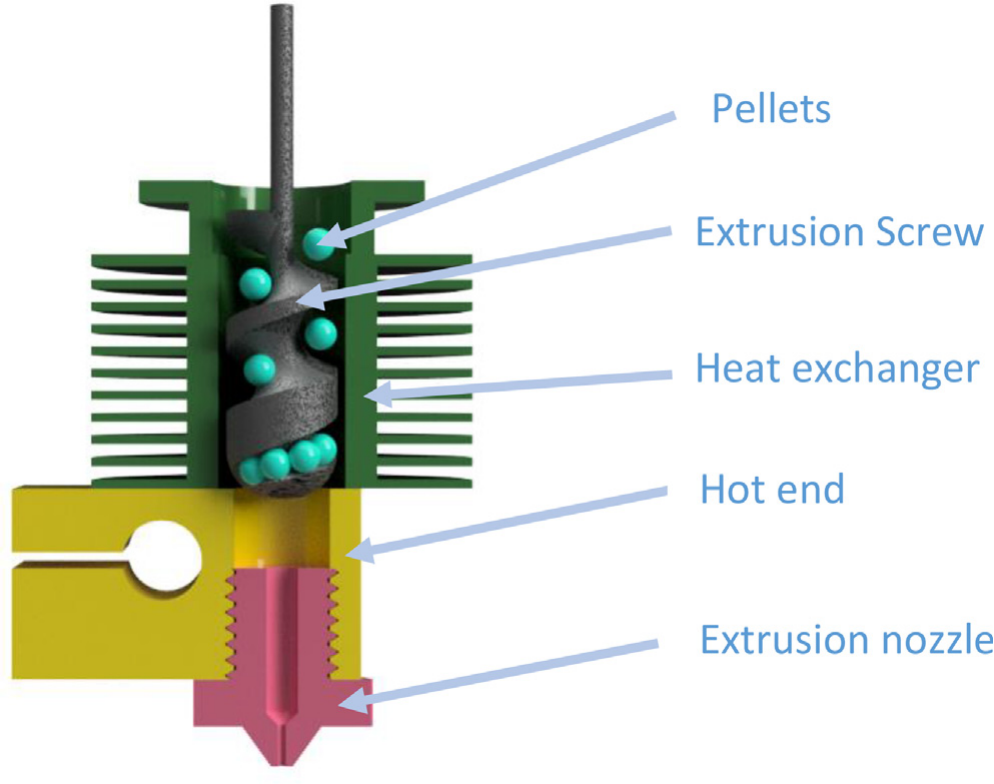
Schematic view of the pellet extruder.

The structure used is composed of four elements that are presented by the diagram in Fig. 1. As for the classic FFF process, we find a heating unit (in yellow) and an extrusion nozzle (in pink), which allow respectively the melting and the extrusion of a rod of polymer + filler mixture to a given diameter.

The material is driven by an Archimedean extrusion screw (in grey), which enables the conveying of the pellets. It is the rotation speed of this element and the implied increase in pressure of the viscous material that dictate the flow of extruded material. Finally, a heat exchanger and a conveying pipe (in green) ensure the dissipation of heat to avoid premature melting of the pellets at the entrance of the extrusion screw and the formation of a plug.

### Creation cycle of a part

The objective of the complete process is to shape parts with complex geometries in a material of metallic or ceramic nature. After thermal post-treatment, the final part will be composed of the target material only and will not contain any polymer.

**Fig. 2:**
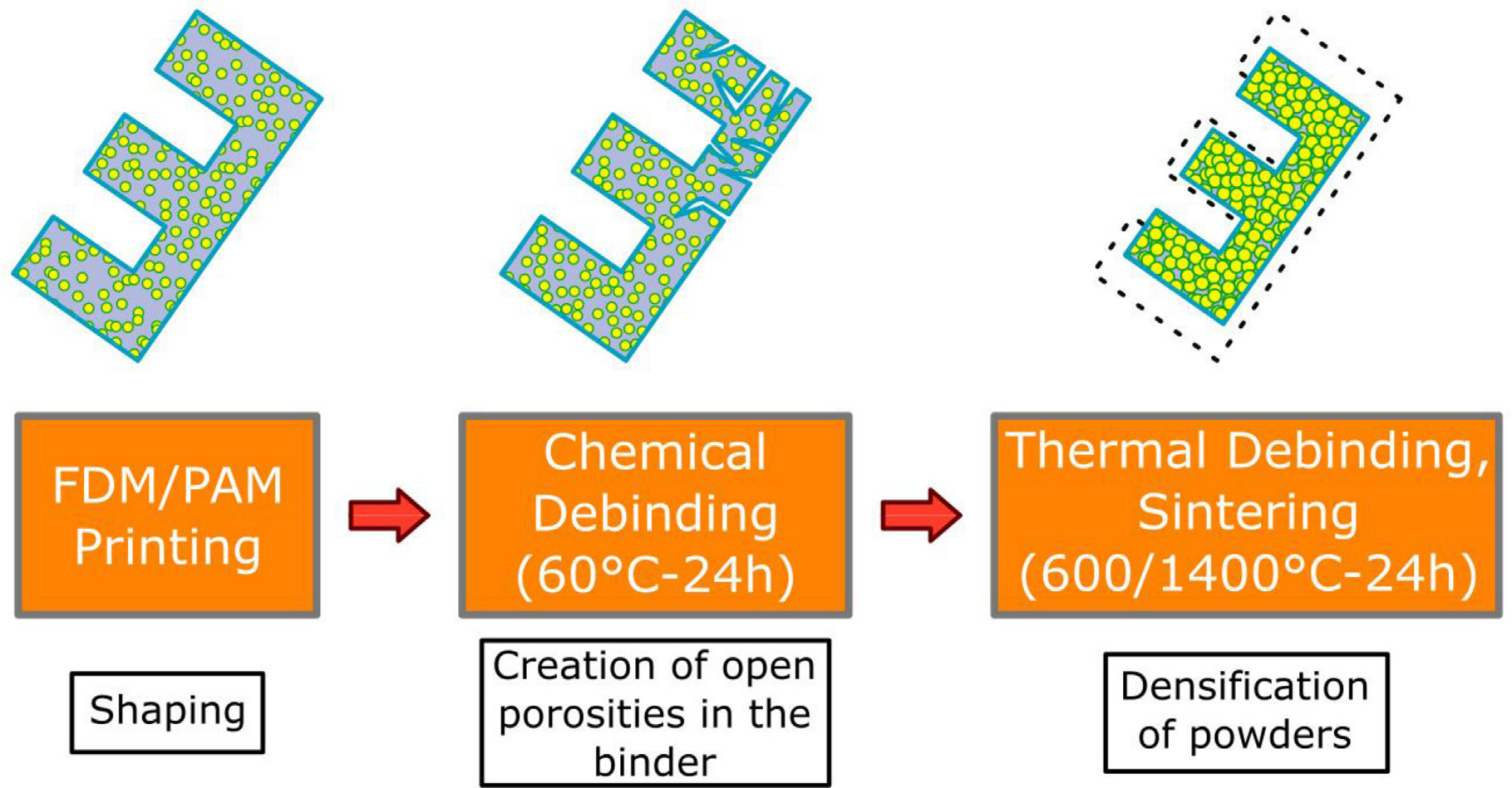
Complete process for the additive manufacture of metal objects from pellets.

Therefore, printing is the first step, which allows to go from the granulates to the “green” part (this term refers to the printed object made of a mixture of the target material in the form of powders encapsulated in the polymer matrix). Then, a part of the polymer binder must be removed during the second step of chemical debinding. This results in the “brown” part. This treatment aims to solubilize part of the polymer matrix in order to create a network of open porosities, which allows the evacuation of the rest of the polymer in the form of gas [61] during the third and last stage: the thermal debinding and sintering. By performing a slow temperature rise, the remaining binder polymer present in the brown part is destroyed. This step is accelerated thanks to the previous chemical debinding, especially for thicker parts. Finally, the last stage of the thermal treatment allows to join the powder grains together in order to densify the part by sintering at high temperature. The complete cycle is illustrated in Fig. 2.

At the end of this cycle, the part obtained is purely metallic (or ceramic if a different powder is used). The density and the porosity state depend mainly on the sintering step (temperature reached, duration of the steps, heating ramps, control of the atmosphere) but also on the quality of the feedstock and the deposition strategy during the printing.

## Hardware description

The mechanical base used to build the system is the Tool Changer platform sold by E3D (see link in Table 2). It is a frame designed for 3D printing and CNC applications (Computer Numerical Control) that has the specificity of being able to use different tools with a single kinematic assembly. For this purpose, a tool holder moves in the horizontal plane and can pick up different modules in four distinct locations. These modules can be used in turn during the same manufacturing job.

The main advantage of this gantry is its great hardware and software accessibility. For its electronic part, it uses a Duet 3D^13^ open source control board whose parameters and source codes are all accessible and modifiable. Moreover, the firmware of this board offers to the user a great control as well as all the necessary elements to allow the development of a non-standard tool quite easily.

To achieve our goal, we reserved one of the tool slots for a modified extruder head capable of printing from granular feedstock as presented above. For this, we chose to use a commercially available extruder, the Pellet Extruder V3 manufactured by Mahor.XYZ (see link in Table 2).

### The E3D tool changer printer

**Fig. 3:**
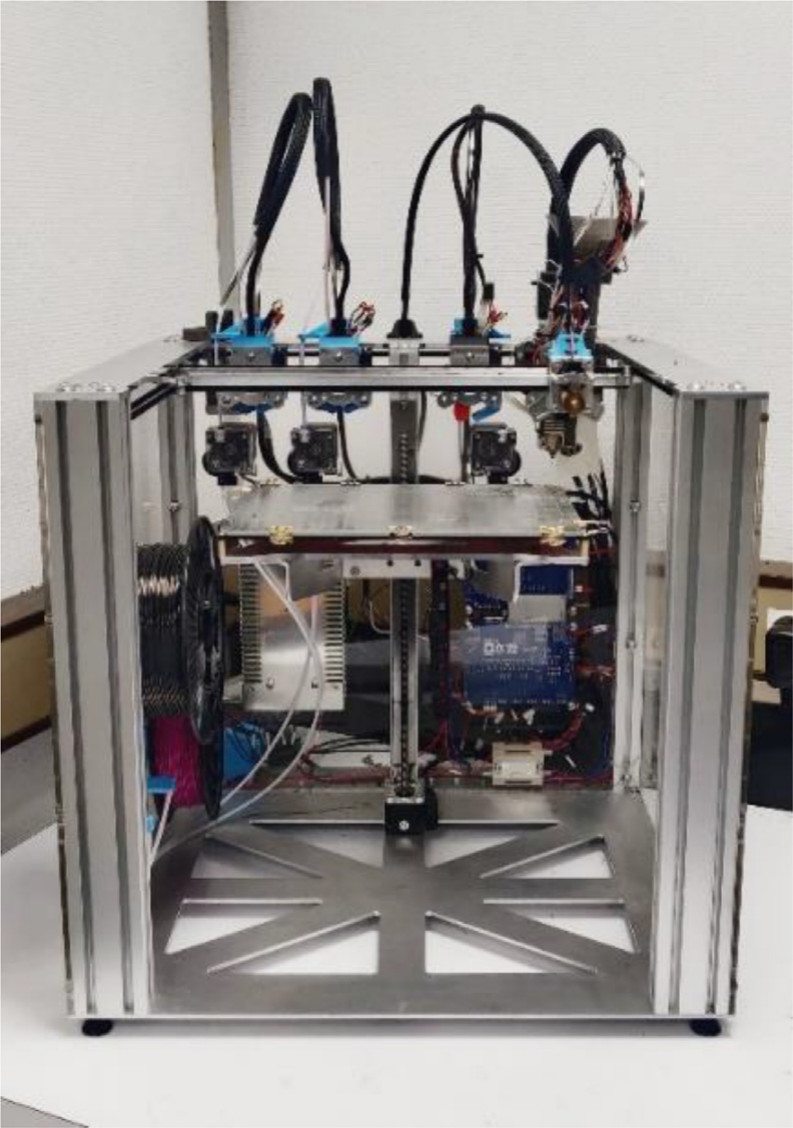
E3D Tool Changer printer.

The Tool Changer printer, seen in Fig. 3, features four similar FFF extrusion heads in its default configuration, allowing for multi-material printing from four filament spools. However, this machine is also meant to bring together different processes to offer a hybrid additive and subtractive manufacturing method. For example, by using 3D printing and CNC machining for the same part. For this purpose, the print heads can be replaced by different tools that will need to be adapted to the frame and the tool holding system. In addition, all information concerning the components’ specifications is provided by the manufacturer. Therefore, on top of being a very robust mechanical unit, this platform is well suited for the development of a tool dedicated to granular printing.

To continue, the machine is equipped with a glass heating plate capable of reaching a temperature of 200 °C and that moves along the vertical axis. Following the same principle, a “core XY” driveline allows to relocate the motors outside the frame and to reduce the inertia of the moving parts. This comes in handy when dealing with larger and heavier tool units.

### The tool-changer head

**Fig. 4:**
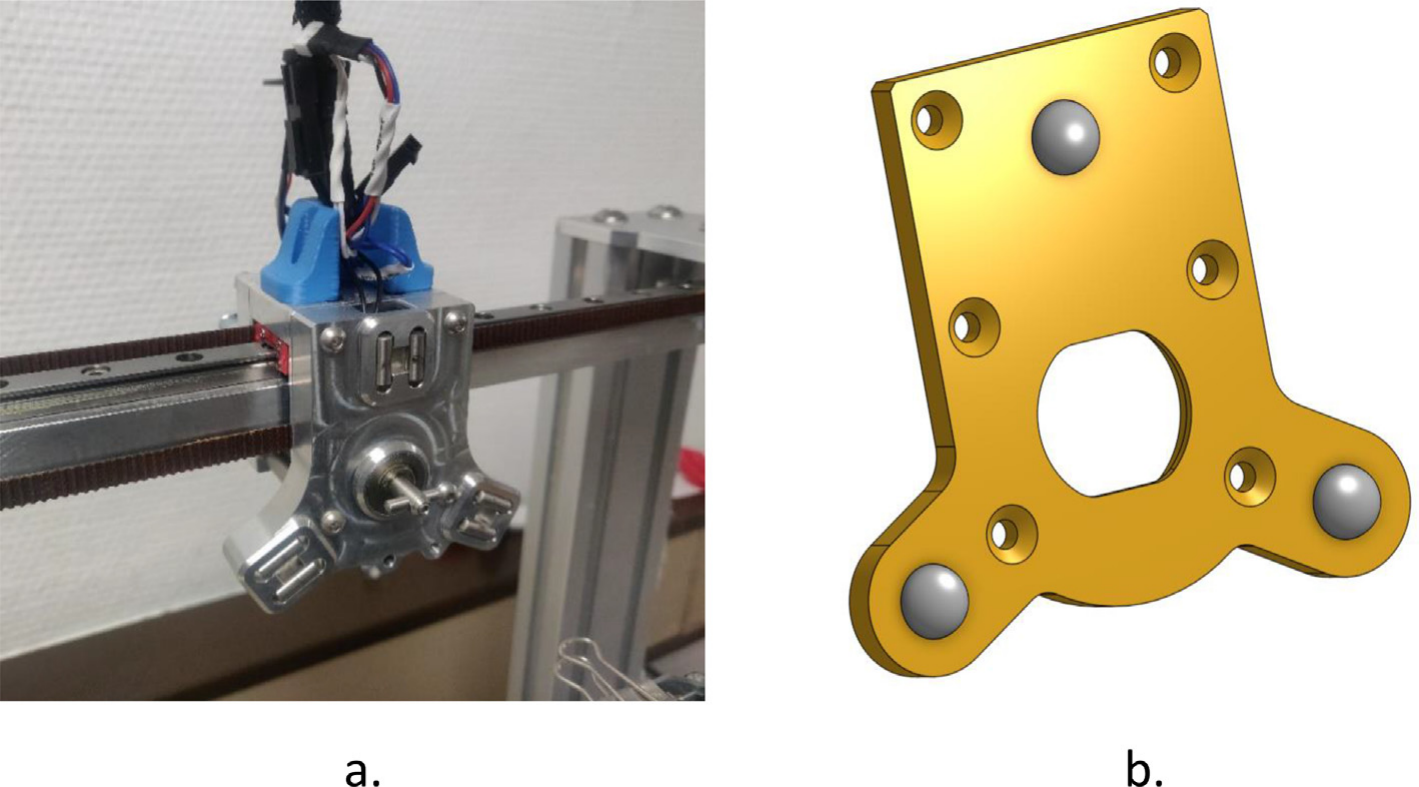
a: Tool holder; b: Mounting plate.

This is the main moving part that allows the various tools to be picked up. It is composed of a T-shaped latch, whose rotation is controlled by a stepper motor, and of six cylinders which, once in contact with the spheres of the fixing plate, form three sphere-cylinder mechanical links (see Fig. 4.a). This assembly moves in the horizontal XY plane.

The Tool Changer carrier is made to fit into the fixing plate (Fig. 4.b) present on each tool and that allows its locking. As said before, this part ensures the positioning of the tool by completing the three mechanical connections, which constitutes an isostatic joint with the tool holder. After insertion, it is held in place by rotating the T-shaped latch by 90° through a slot placed facing it in the fixing plate.

It is this system that allows the system to repeatedly place and remove tools and that ensures positioning accuracy. Aside from sizing each element to fit the mounting plate, only the total weight of the module must be taken into account so that the latch spring can hold the whole assembly.

### The Mahor XYZ extruder

**Fig. 5:**
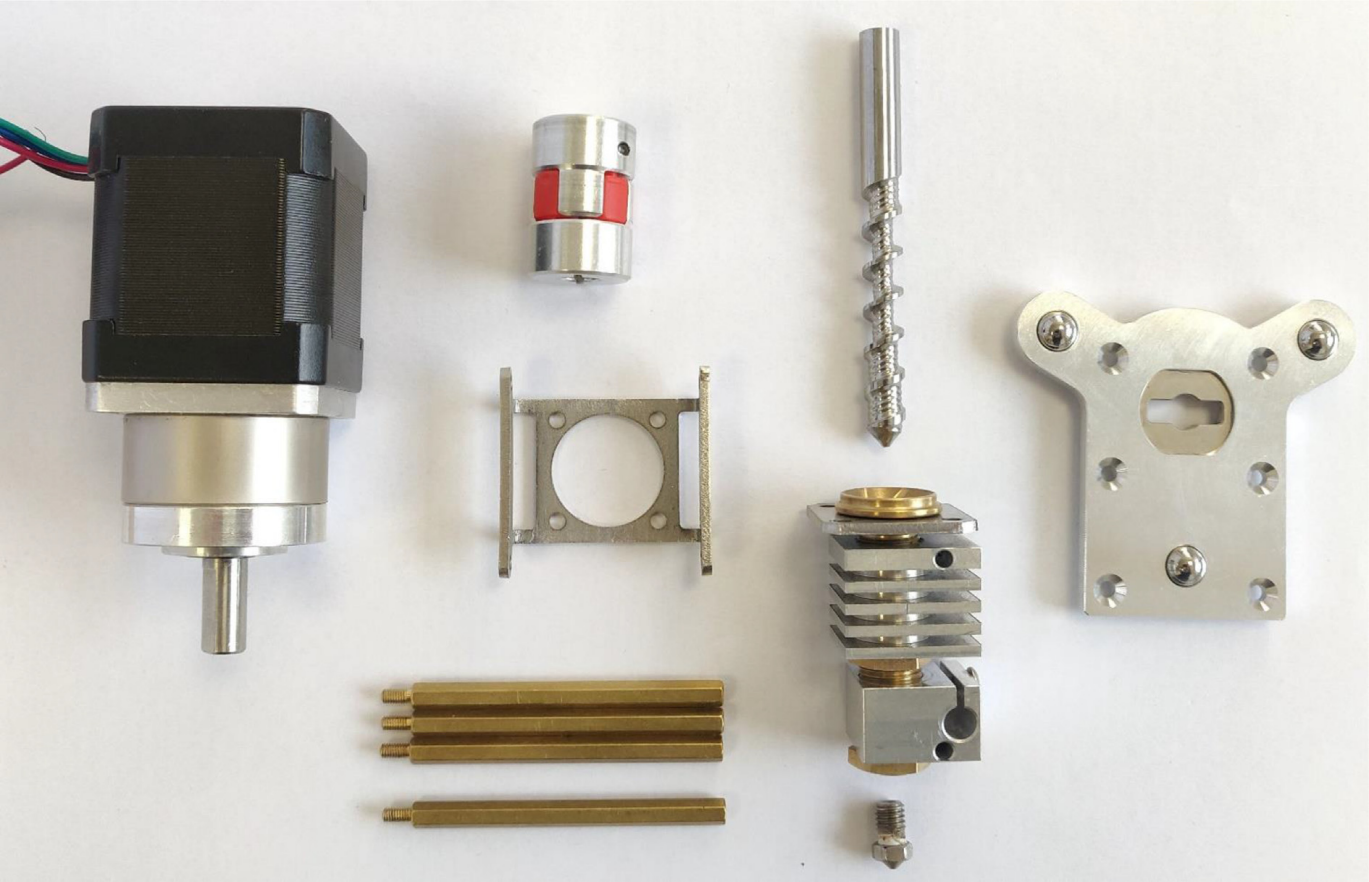
Mahor V3 Extruder parts + E3D fixing plate.

**Fig. 6:**
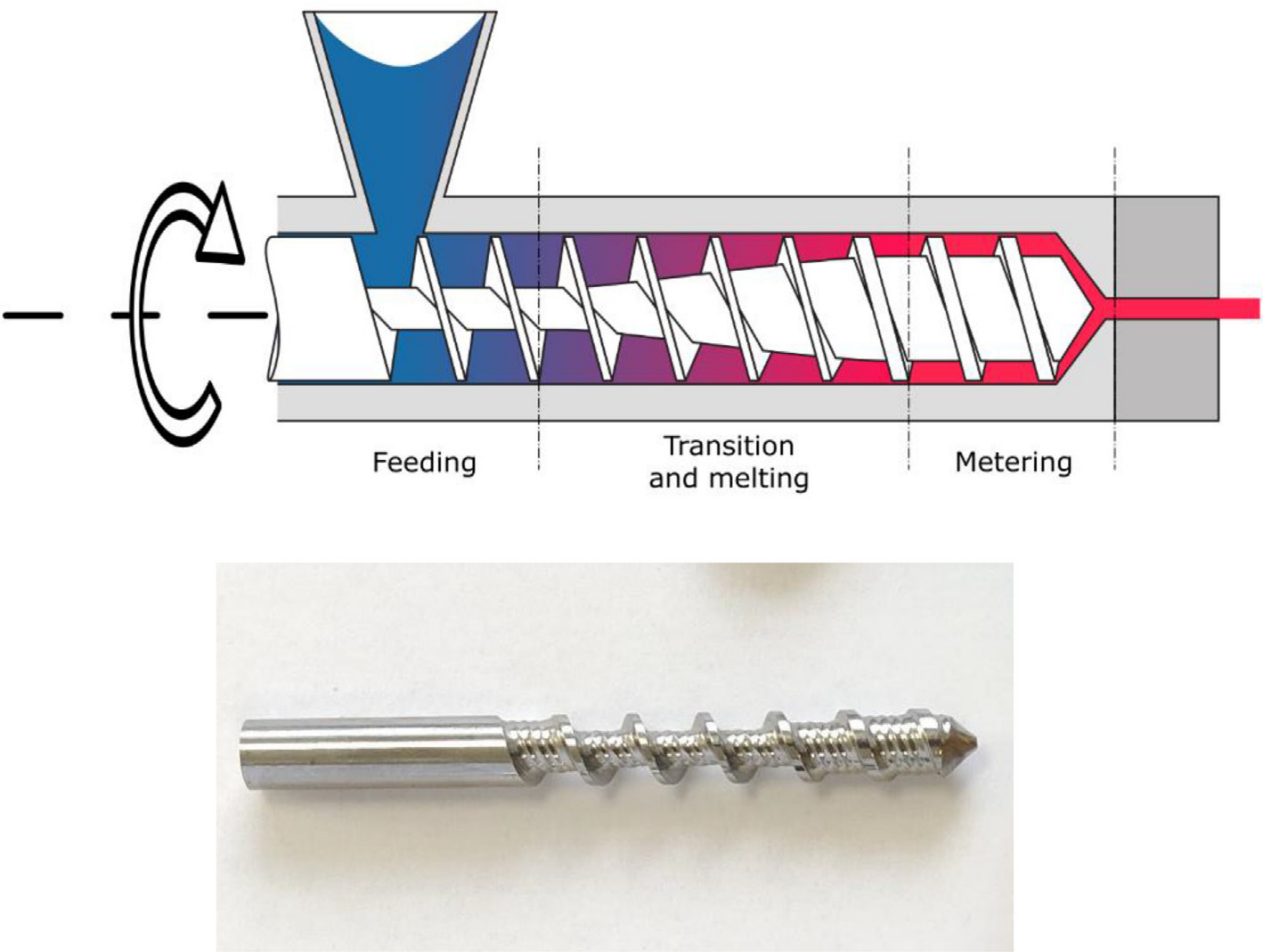
Extrusion screw typical design.

The Mahor XYZ V3 extrusion head is used to perform the printing of material from a feedstock in the form of pellets. It is a component sold on its own by Mahor.XYZ that mainly consists of an extrusion screw, a stepper motor and a hot end (see Fig. 5). The temperature of the extruder is controlled by a heating cartridge, which ensures a sufficiently fluid molten feedstock at the extrusion nozzle, and by a fan whose rotation speed allows to control the temperature gradient along the extrusion screw. The temperature is measured at two points: directly in the hot end, next to the heating resistor, and at the top of the screw’s casing heatsink next to the pellet inlet. (See Fig. 6.).

A small extrusion screw is used to convey and melt the pellets. Above it, a reservoir with an additional fan allows the granules to be stored and kept cool until delivered. This ensures that the polymer part of the pellet remains solid so that they can fall and reach the screw inlet. The heat from the heating element is transferred by conduction to the screw housing and the extrusion nozzle. By properly adjusting the temperature, the polymer part of the pellets is progressively melted during the conveying and compression process created by the rotation of the screw.

Adaptation parts must be fabricated to fit this tool to the fixing plate presented earlier. These parts will be presented further in the “build instructions” chapter.

#### Extrusion screw

Extrusion screws, also referred to as Archimedean screws, are widely used in thermoplastic polymer applications. For instance, it is the main tool for creating large formulation from blends of different nature of polymer. As stated before, PIM applications also use this extrusion process in order to shape parts into a mold from a granulates feedstock. The system of the Mahor V3 merely does the same in a smaller form factor except that the extruded rush is not injected but orderly deposited to create a shape.

The shape of the extrusion screw and its enclosure are designed to improve the conveying and control of the material flow [62], [63]. It is composed of three sections: the feeding zone, the transition and the metering zone.

The profile of the screw exhibits a reduction of the available volume in the transition section. This shape creates an increase in pressure and thus enables the pellets to be crushed and air bubbles to be expelled. The result is a homogeneous material string at the outlet of the extrusion head. Similarly, the entry part of the casing of the screw features helicoidal stripes that increases its friction factor and the conveying of the pellets.

A similar extrusion screw is set in the case of Mahor extrusion system. The modeling proposed by the previously quoted bibliography gives a linear relation between the extrusion flow and the rotational speed. However, early tests showed that the control of this material flow is a critical point in order to obtain a successful print. Typical deposition problems are encountered when printing small details where the speed quickly changes and the quantity of extruded material is lagging behind the movement of the print tool. Strategy to overcome this problem have been implemented in the RepRap firmware G-code control and will be presented in the operation instructions section.

### 3D Duet board

**Fig. 7:**
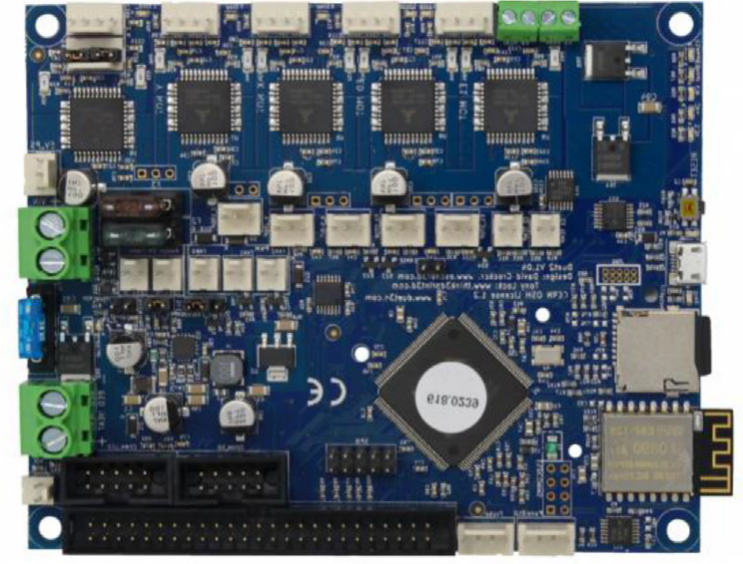
Duet 2 Wifi Board [26].

The E3D Tool Changer printer is equipped with a “Duet 2 WIFI” control board, sold by the company Duet3D (Fig. 7). This is an open hardware and open source board designed to control 3D printers, which has the advantage of giving considerable control to the user. Its large number of connections offers the possibility to connect several components that are essential for the development of our pellet printing system. In particular, G-code controlled 24 V PWM (Pulse Width Modulation) power supplies for additional fans as well as a second temperature sensor.

The software part of the board is provided by the Reprap firmware^14^. Its main strength is that it is fully configurable in G-code language, which allows for quick modification of machine parameters, whereas other solutions such as Marlin require the board to be restarted or the configuration file to be recompiled. Thanks to this, several advanced functions offered by the G-code become more flexible and easier to use. Examples include customizing the positioning for each tool via dedicated offset variables, or modifying the extrusion setpoint using a linear feed-rate multiplier based on acceleration (“pressure advance”) or a non-linear speed setpoint. The full G-code control also allows the creation of macros that can be called at any time during printing and that automate predefined series of instructions. For example, they are used by default by the Tool Changer printer to carry out the series of movements that are used for the seizure of a head by the tool holder or those that can help to improve the printing of granules.

The control of the printers could be further improved with the newest version of the board available that could include higher currents capabilities as well as CAN bus connectivity in order to interface with a wider range of actuators such as different extruders or machining tools.

To summarize:

• The base hardware used here offers a very sturdy and open framework, both mechanically and software wise, to develop non-conventional deposition printing technologies and others.
• The versatility of the printer's gantry and the possibility of changing the tool allow to perform classical Material Extrusion additive manufacturing tasks, useful for the adaptation of the extrusion head, while offering an important diversity of materials thanks to the feedstock in the form of granules.
• The whole densification process, inherited from the PIM process, allows to additively shape metallic objects at a fraction of the cost required by SLS or SLM methods.

## Design files

### Design files summary

(See Table 1).

**Table 1:** List of the necessary printable components.

**Design file name**	**File type**	**Open source license**	**Location of the file**
*TC-Dock_L*	CAD file	*Creative Commons Attribution-ShareAlike license*	
*Fan_Holder*	CAD file	*Creative Commons Attribution-ShareAlike license*	
*Pellet case*	CAD file	*Creative Commons Attribution-ShareAlike license*	
*Cooling pipe*	CAD file	*Creative Commons Attribution-ShareAlike license*	
*Funnel*	CAD file	*Creative Commons Attribution-ShareAlike license*	

Each part listed above is presented in the “Build instructions” section as well as its utility and how they should be used in the assembly.

**Table 2:** List of required parts.

**Designator**	**Component**	**Number**	**Cost per unit -currency**	**Source of materials**	**Material type**
*3D Printer*	*ToolChanger & Motion System Bundle*	*1*	*£ 1854*	https://e3d-online.com/products/toolchanger-motion-system-bundle	*-Other*
*3D Printer*	*Blank Tool Plate & Dock Kit*	*1*	*£ 56*	https://e3d-online.com/products/blank-tool-plate-dock-kit	*-Other*
*Pellets Extruder*	*V3 Pellet Extruder*	*1*	*€ 500*	https://mahor.xyz/producto/pellet-extruder-v3/	
*3D Printing Filament*	*SpoolWorks Edge Filament*	*1*	*£ 25*	https://e3d-online.com/collections/filament/products/spoolworks-edge-filament	*-Polymer* *→PETG*
*Screws*	*M3, length: 8 mm, conical head*	*4*	*Included with the E3D Tool Kit*		*-Metal*
*Screws*	*M3, length: 11 mm*	*4*	*Included with the Mahor V3 Pellet Extruder kit*		*-Metal*
*Screws*	*M3, length: 15 mm*	*5*	*€ 26, box of 200*	https://fr.rs-online.com/web/p/vis-a-six-pans/8229063	*-Metal*
*Screws*	*M3, length: 20 mm*	*2*	*€ 23, box of 50*	https://fr.rs-online.com/web/p/vis-a-six-pans/0293319	*-Metal*
*Screws*	*M3, length: 10 mm*	*4*	*€ 16, box of 100*	https://fr.rs-online.com/web/p/vis-a-six-pans/1838604	*-Metal*
*Washer*	*Diameter: 12 mm*	*2*	*€ 8, box of 100*	https://fr.rs-online.com/web/p/rondelles-plates/0189658	*-Metal*
*Washer*	*Diameter: 9 mm*	*2*	*€ 6, box of 100*	https://fr.rs-online.com/web/p/rondelles-plates/0189636	*-Metal*
*Fans*	*24 V fan*	*2*	*€ 1*	https://winsinn.com/40mm-fan/	*-Other*

## Bill of materials summary

After conversion, the total price of the material investment necessary for the construction of this equipment is two thousand six hundred seventy-six euros. Note that this doesn’t include the equipment used for the assembly. Necessary tools are listed in Table 3.

**Table 3:** Neccesary tools.

**Necessary tools**
M3 Allen key
Small pliers
Soldering iron
Soldering tin wire
Heat-shrink tubing

## Build instructions

### Assembly of the E3D tool changer

The prior assembly of the Tool Changer printer is of course necessary. This part is not addressed here since it is already very clearly presented by the E3D team, which is the originator of this motion system. Every useful information can be found in a series of documents^15^ on E3D site and detailed assembly and configuration instructions have been put on video by René Jurack^16^.

#### Adaptation of the Mahor extrusion head on the tool changer

One of the strengths of the E3D Tool Changer is its software and hardware openness. The user is given geometric models in STEP format of the main parts such as the tool holder and the coupling plates, which facilitates the design work to be able to adapt and mount a new head. The RepRap firmware version used on the Duet 2 WIFI board allows the user to easily modify the carrier head behavior for each head. For example, by specifying slower travel speeds for heavier tools or by adjusting the offset between the tool holder and the extruder nozzle position.

In this section, the parts that are used to mount the Mahor XYZ extrusion head with the E3D Tool Changer system are described. These parts are 3D printable by the printer itself or any other FFF or DLP solution. These prints require very little post-processing before they can be assembled.

##### Part presentation

###### L-shaped docking connector. (« TC-Dock_L »)

This part is an adaptation of the “TC-Dock” component that equips the “bowden” filament printing heads (“E3D V6″) supplied with the purchase of the tool changer. The design proposed by E3D fulfills two functions: it ensures the connection between the fixing plate (”TC-Plate“) and the rest of the print head or the tool; it also serves as a docking point to store the assembly on the frame of the gantry once it has been deposited by the tool holder.

**Fig. 8:**
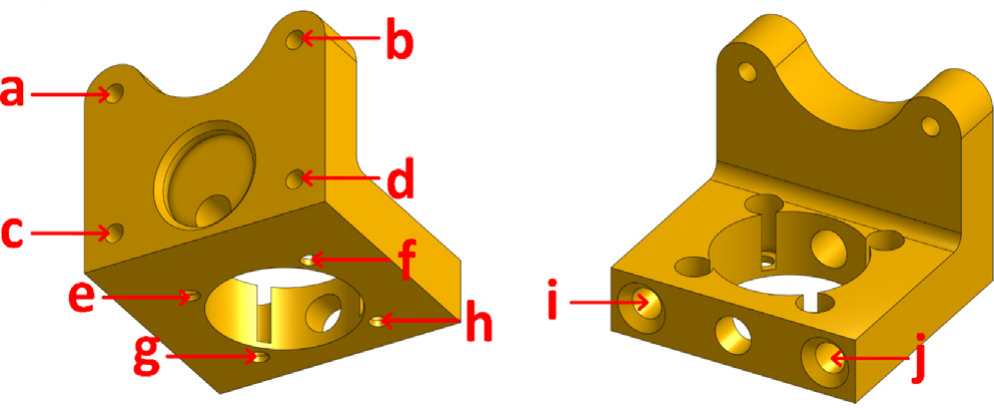
“TC-Dock_L”.

The component that we made, named “TC-Dock_L” and presented in Fig. 8, includes the functions of the original part and is adapted to the shape and dimensions of the pellet extrusion head. In addition, the part has been thickened at the point of attachment with the Mahor extruder and a bracket has been added to accommodate two additional screws as well as to stiffen the assembly. Indeed, since the entire weight of the tool, which is greater than that of conventional extruders, is supported by this element, the modifications described here ensure the integrity of the assembly, even though it is a printed part.

###### Fitting and post processing

A difficulty that is often encountered when assembling printed parts is getting the correct fits. Depending on the nozzle used, the material or the printing parameters, it may be necessary to correct certain holes by conventional drilling or by using a fine file and sandpaper.

The TC-Dock_L part has four holes (marked a, b, c and d in Fig. 8) that must be tapped with an M3 thread tap to screw in the mounting plate. To allow tapping, the diameter of these holes must be 2.5 mm. Four other holes (marked e, f, g and h in Fig. 8) extend in the same axis the blind holes that accommodate the hexagonal spacers of the extruder. These holes must be slightly larger (3.3 mm), so as not to interfere with the passage of the spacers' thread. Finally, the two holes (i and j in Fig. 8) in which the support pins are inserted are used to support a tool that has been dropped off. They must allow a perfectly sliding movement and their diameter, initially equal to 4.8 mm, may also have to be slightly enlarged after printing.

###### « Fan_Holder »

In order to make printing from granules work, the creation of a temperature gradient along the screw casing is necessary. This is primarily to keep the granules in a solid state upstream and just at the beginning of the screw in order to avoid the creation of a plug. For this purpose, a small fan (“Heat-Sink Fan”) blows on the radiator already present on the Mahor extrusion head and which surrounds the screw barrel. In addition, to improve the printing results, a second fan (“Part Cooling Fan”) is often used to cool the rod at the nozzle outlet in order to quickly solidify it.

**Fig. 9:**
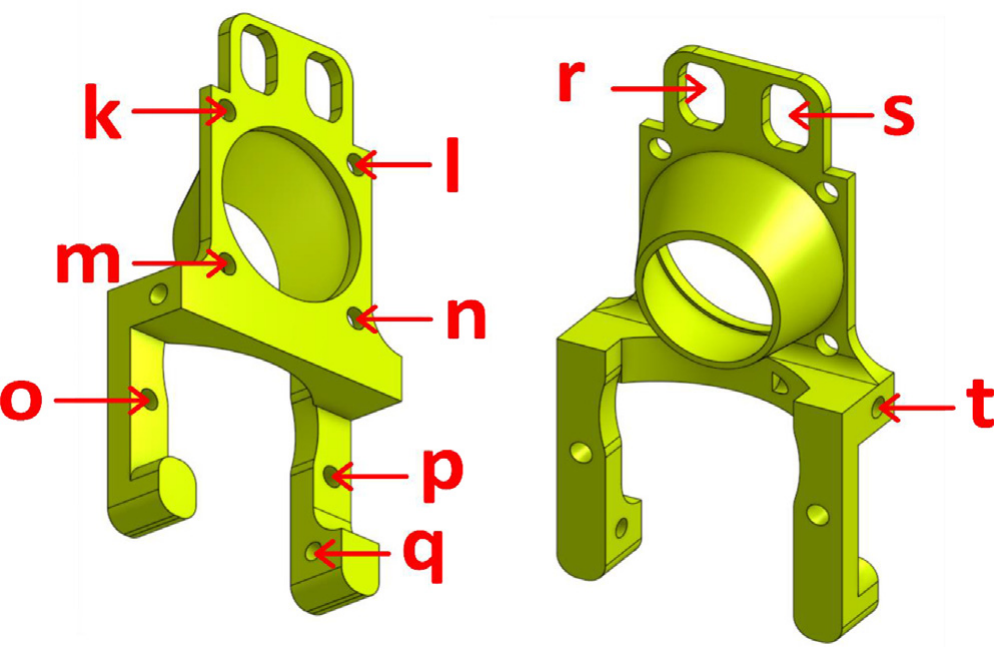
“Fan Holder”.

The “Fan_Holder” part, visible on Fig. 9, supports these two fans and is attached to the tool with two screws. The holes o and p must therefore be tapped to M3 dimensions, as well as holes k, l, m and n, which are used to attach the “hot-end” fan. The part also incorporates a duct that is intended to concentrate the airflow from the latter onto the radiator. Finally, the “Pellet cooling fan” is held to this part by a single screw in the hole q. In the same way, the “Part Colling” fan is held by a screw in the hole t. To direct the air flow of the latter, a cooling pipe must also be printed and fixed on the fan holder. It should be adjusted in height thanks to the two oblong holes r and s.

###### Funnel and Pellet Case

**Fig. 10:**
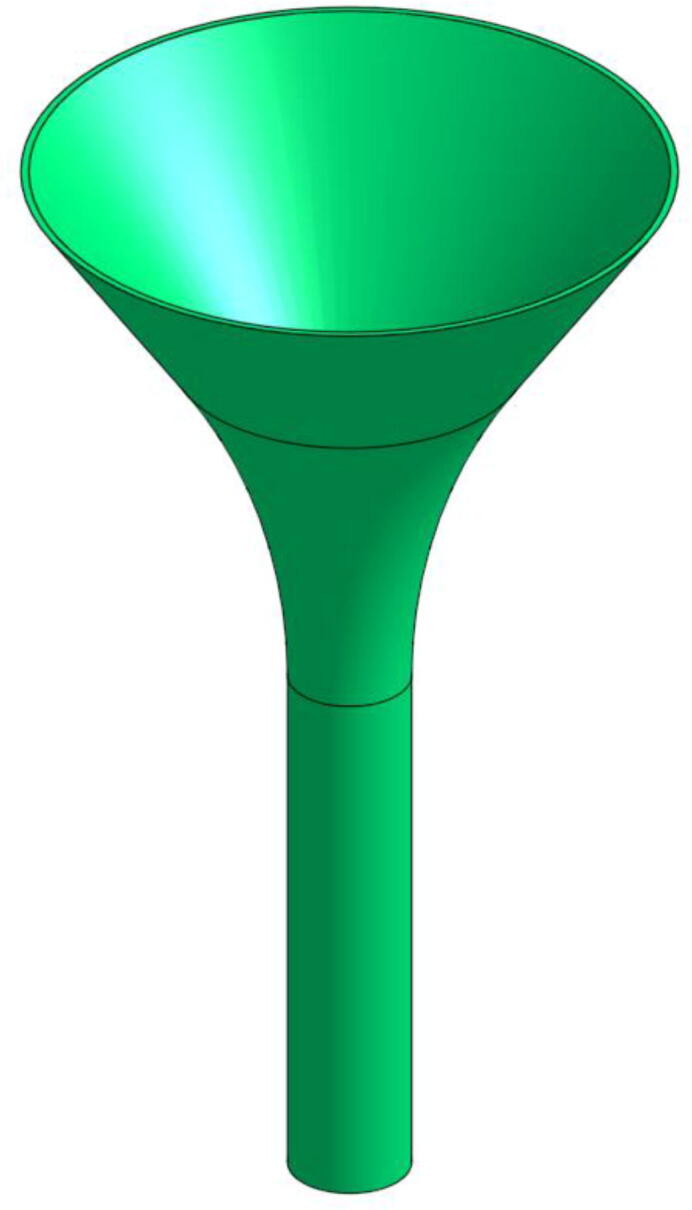
Funnel.

To store the quantity of granules required for a print run, a simple funnel with the dimensions of the pellet extrusion head can be printed (Fig. 10). The proposed geometrical file allows the user to have a storage capacity of about 20 cl. This funnel fits into a second part called “Pellet Case”.

**Fig. 11:**
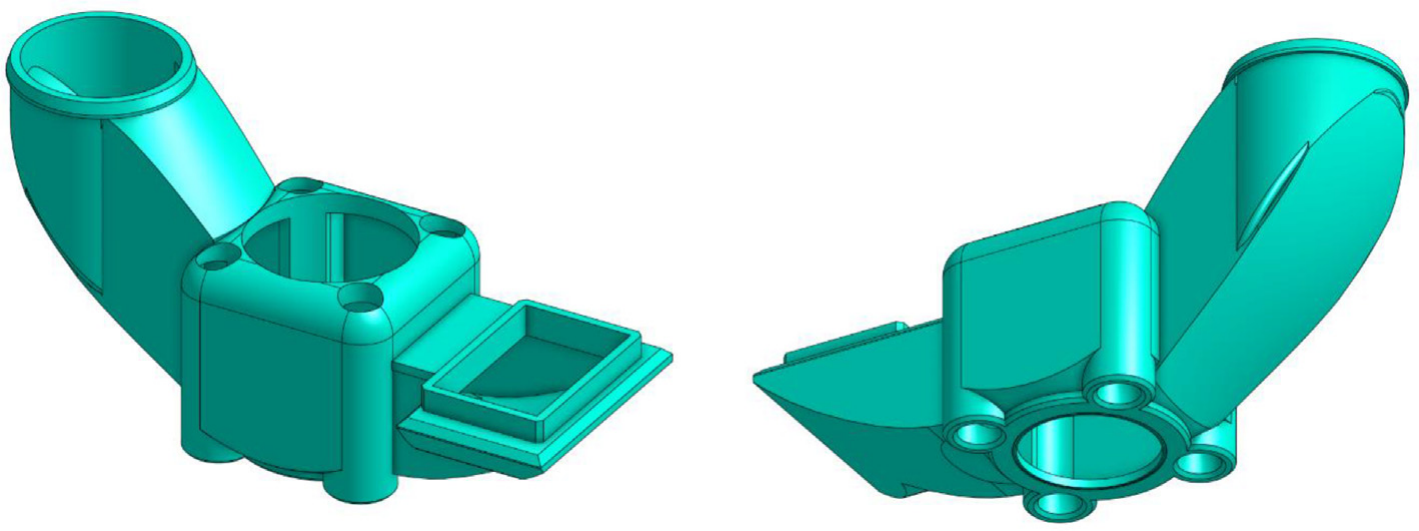
“Pellet Case”.

The latter ensures that the pellets are conveyed to the beginning of the extrusion screw (Fig. 11). A design version of this component is provided by Mahor XYZ. It has two inlets, one for the pellets to reach the screw and the other to allow a fan to blow on the pellets to cool them. Just as presented previously for the “Heat-Sink Fan”, the purpose of this is to prevent the pellets from melting in the “Pellet Case” so that they do not clog the inlet of the extrusion body.

The modifications made on this component are meant to increase the inclination of the slope that makes the pellets slide and to create walls that completely cover the sockets. The aim is to prevent the pellets from getting stuck as much as possible. Angular pellets whose size is too close to the diameter of the duct are likely to jam, which led to numerous printing errors during the first development tests.

Since this part is in contact with the top of the screw sleeve, it can be subjected to high heat. It is therefore recommended to print it with a higher melting point material such as ABS, ASA or PEEK. In our case, all parts were made of PET, which has a melting point of 245 °C. However, after several hours of use, the part still softens and a slight thickening can be observed at the contact with the heating body. Another solution is to use a DLP printer with a thermosetting resin that will be less sensitive to temperature and will not deform at all.

###### «Cooling_Pipe»

**Fig. 12:**
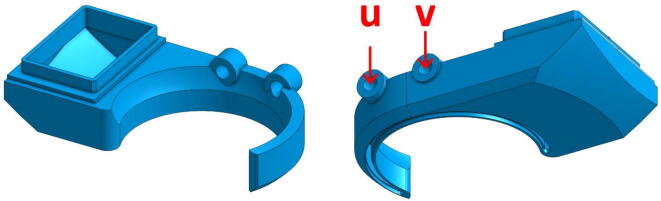
« Cooling Pipe ».

The last part to be printed is a pipe that is used to direct the air flow from the third fan. This one is classically used in 3D printing and is dedicated to cooling the extruded rod right from the nozzle to help with the printing process. The part has two holes, named u and v on Fig. 12, that must be tapped in size M3 to allow mounting on the “Fan_Holder” part. The design of this part, proposed by Mahor, has been modified to work with the rest of the parts shown before. To position it correctly, it must be placed just above the extrusion area, making sure that it does not touch the part of the object that is already printed.

##### Assembly instructions

The assembly of the extruder with the printed adaptation parts and the fixation plate requires 17 M3 screws. Four conical head screws are provided with the Blank Tool Plate & Dock Kit from E3D and four 5 mm long screw are given with the Mahor extruder.

**Fig. 13:**
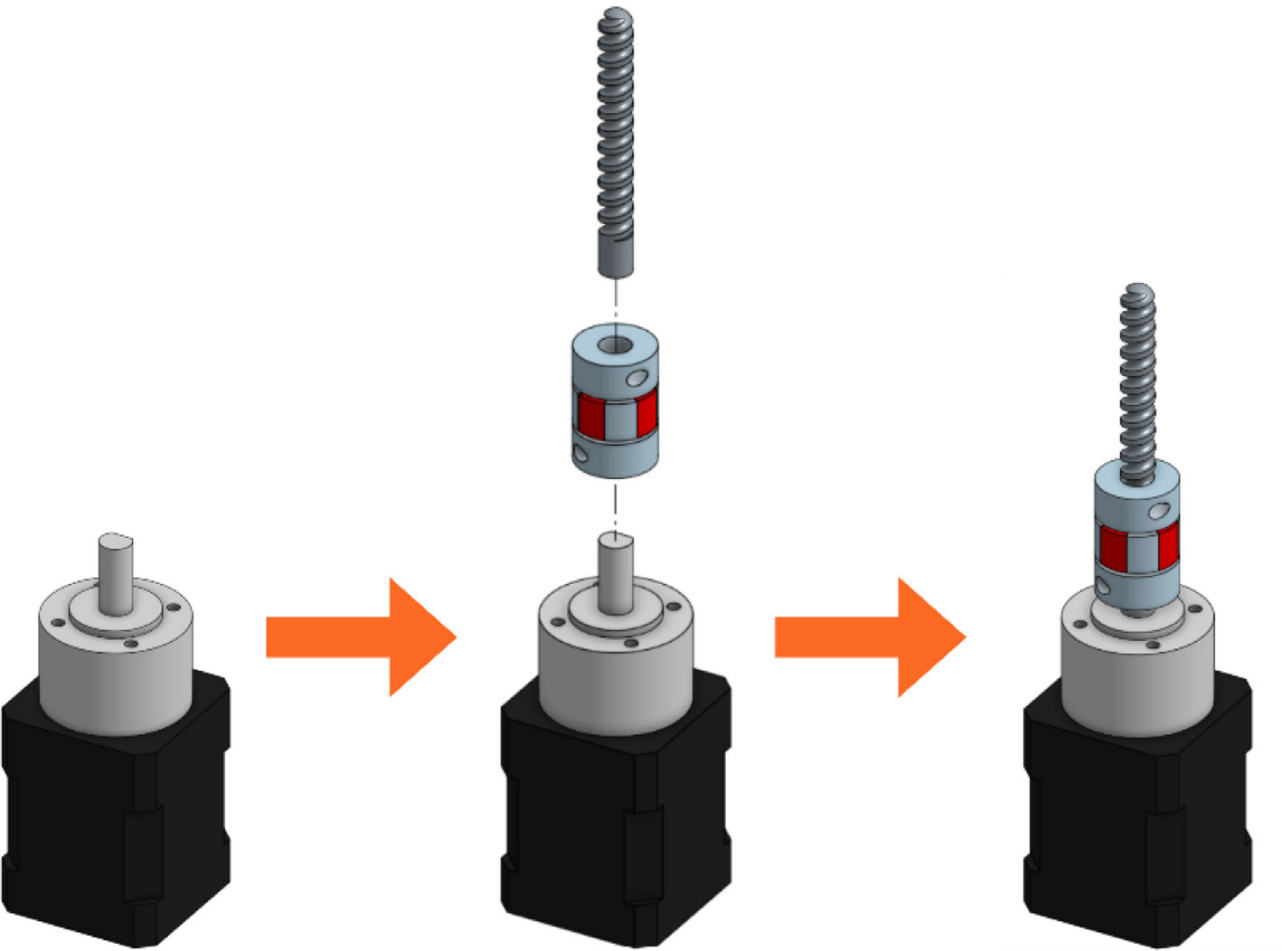
Extrusion Screw and motor coupling.
• A metallic grub screw (included in the E3D blank tool kit) must be fitted in the TC-Dock_L part in order to allow it to be dropped and retained by a magnet. This yellow part must then be held on the motor with four hexagonal spacers on which the Pellet Case is slid as shown in Fig. 14.

In the next figures, the parts that are included with the kit from E3D and the V3 Pellet Extruder package (Fig. 5) are illustrated with gray or black shades. The parts that must be 3D printing (presented above) are represented with different vivid colors to facilitate the understanding of the assembly instructions:

• Mount the extrusion screw on the stepper motor using the coupler (Fig. 13). The coupler is equipped with two clamping screws to tighten each axis. These must be fully inserted into the coupler.

**Fig. 14:**
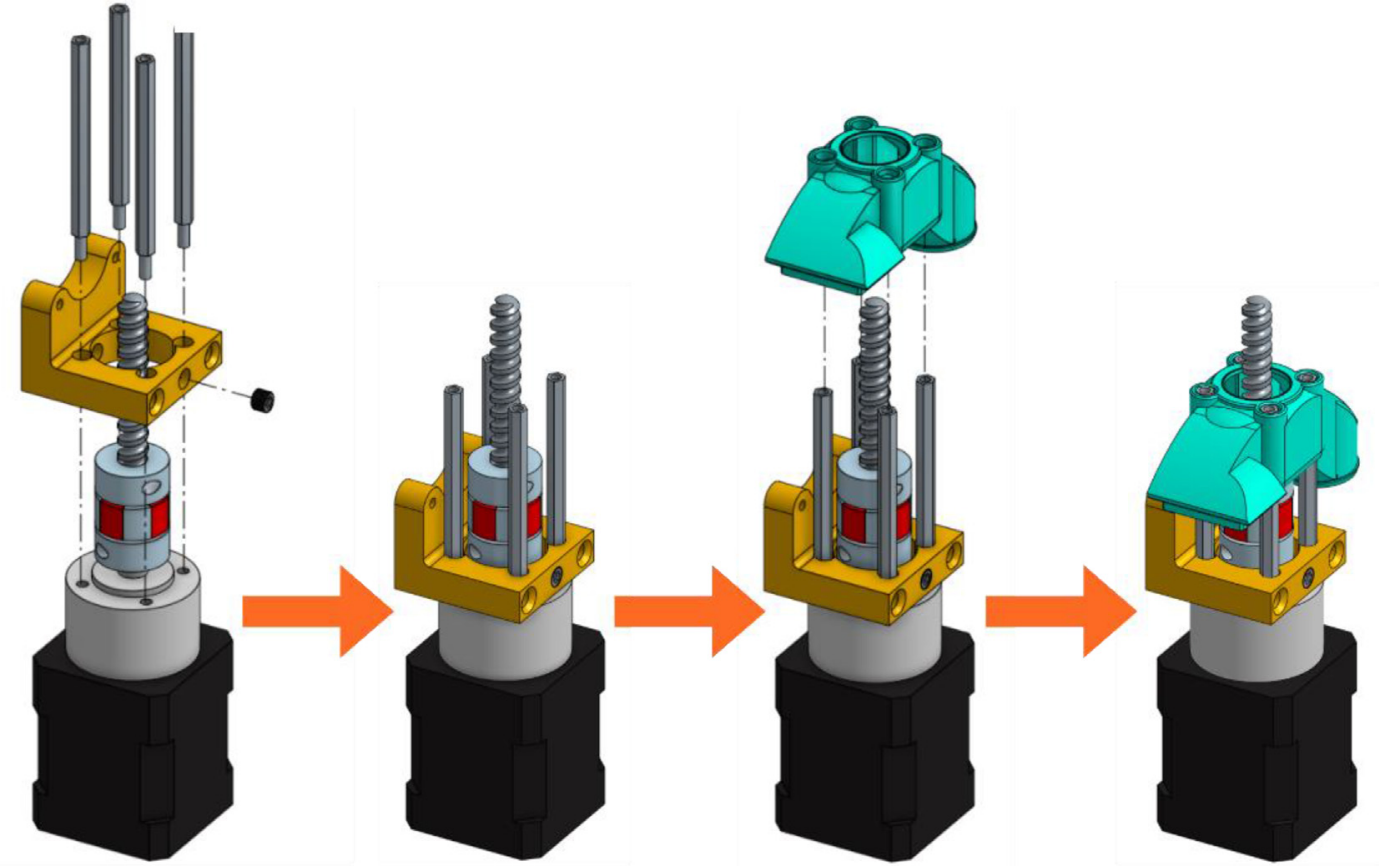
TC-Dock_L and Pellet Case mounting.
• Place the steel mounting bracket and the heating body opposite to the spacers and insert the extrusion screw into the heating body (Fig. 15). The heater must first be turned 45° clockwise to allow the 3 mm long M3 screws to be inserted into the spacers' threads. The screws must not be fully tightened yet.

**Fig. 15:**
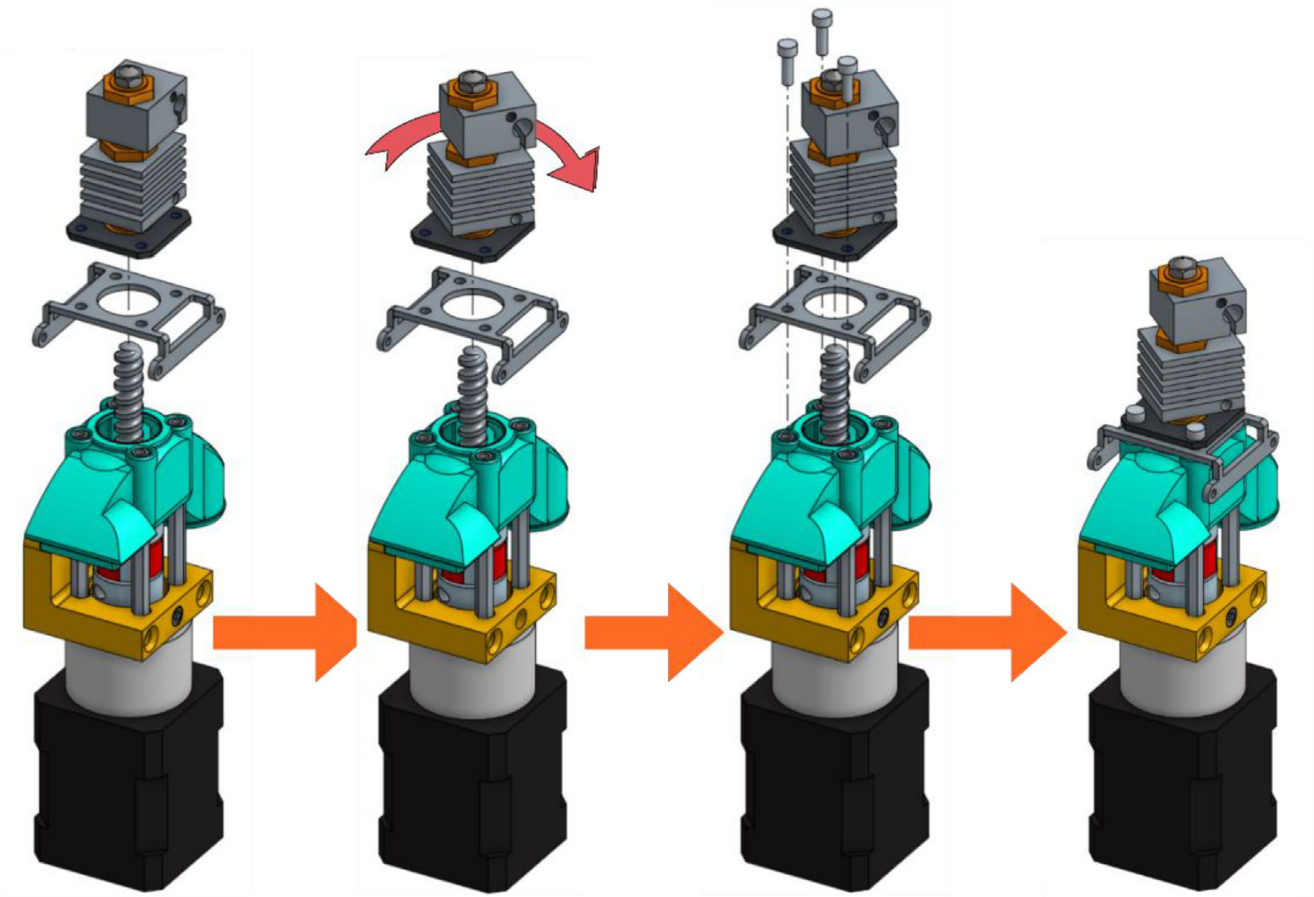
Fixation of the heating body.
• Turn the heating body 90° counter-clockwise in order to insert the fourth screw. This one should be tightened and the heating body turned back to allow the tightening of the three others. Contrary to what can be seen on the last scheme of Fig. 16, it might not be possible to place it back perpendicurlaly to the steel mounting bracket but this wont interfere with the rest of the operation.

**Fig. 16:**
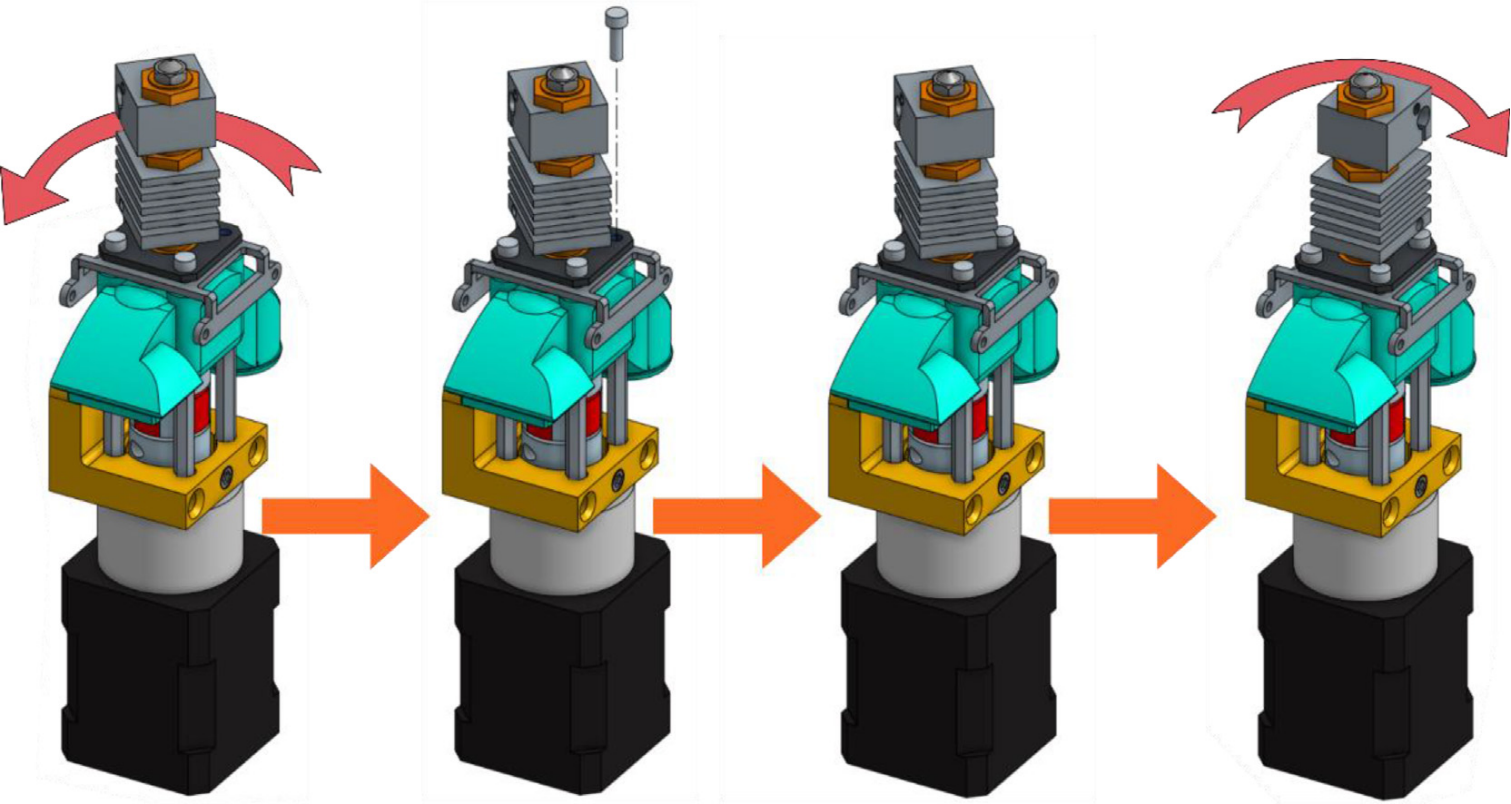
Tightening of the heating body maintaining screws.
• After rotating the whole 90° counter clockwise in order to face the front part of the extruder as seen in Fig. 17, the fan holder must be attached onto the mounting bracket with two 10 mm long M3 screws. To allow an allen key to access the hexagonal pattern, the pellet case must be momentarilly slid down. The screws must be put this way because the holes in the mounting bracket are tapped.

**Fig. 17:**
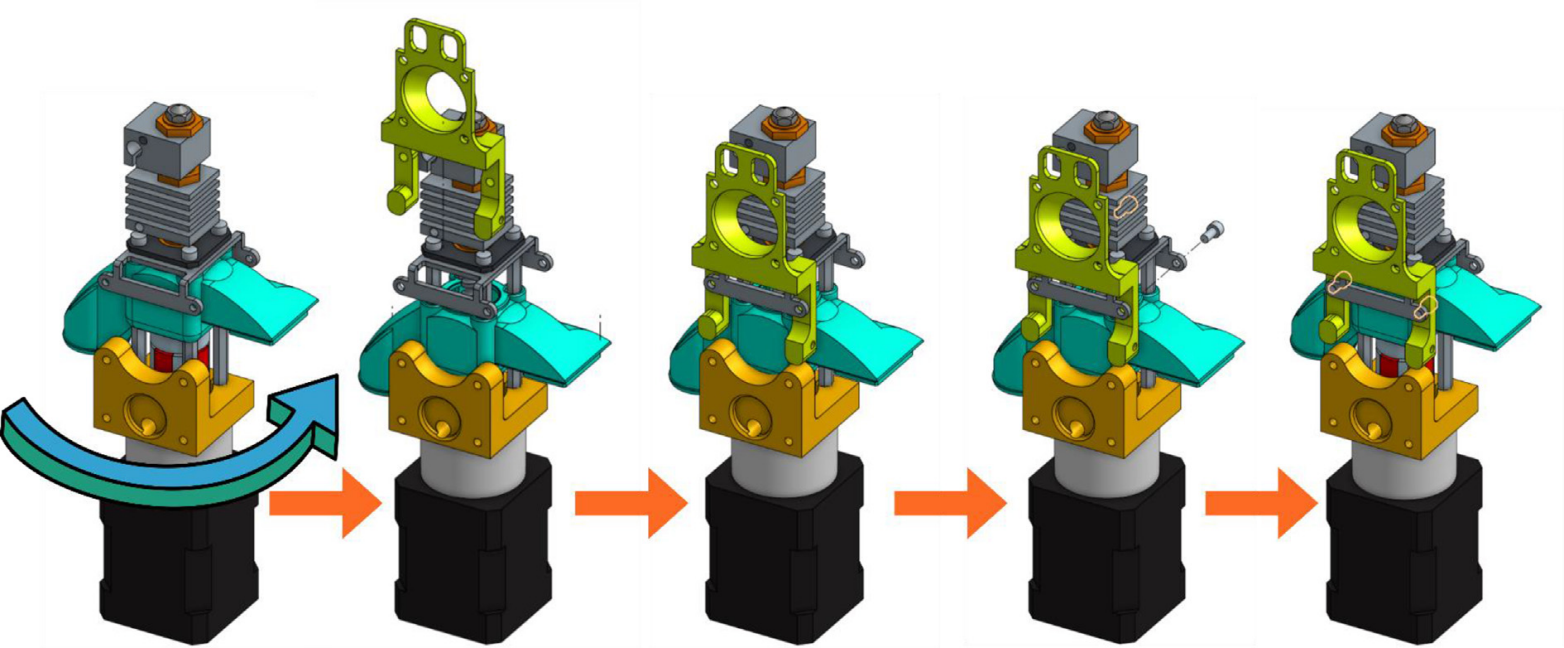
Installing the Fan Holder.
• The fixation plate can then be mounted on the TC-Dock_L using four conical head screws (Fig. 18).

**Fig. 18:**
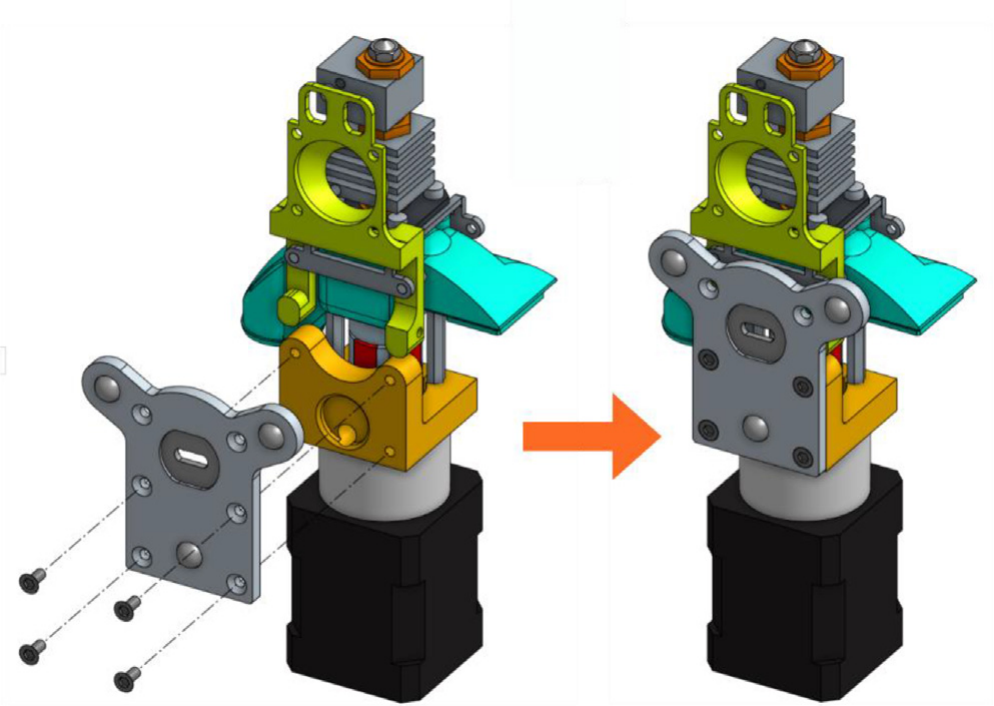
Association of the fixation plate with the docking part.
• The next scheme from Fig. 19 shows how to mount the Cooling Pipe, as well as the Heat-Sink fan.

**Fig. 19:**
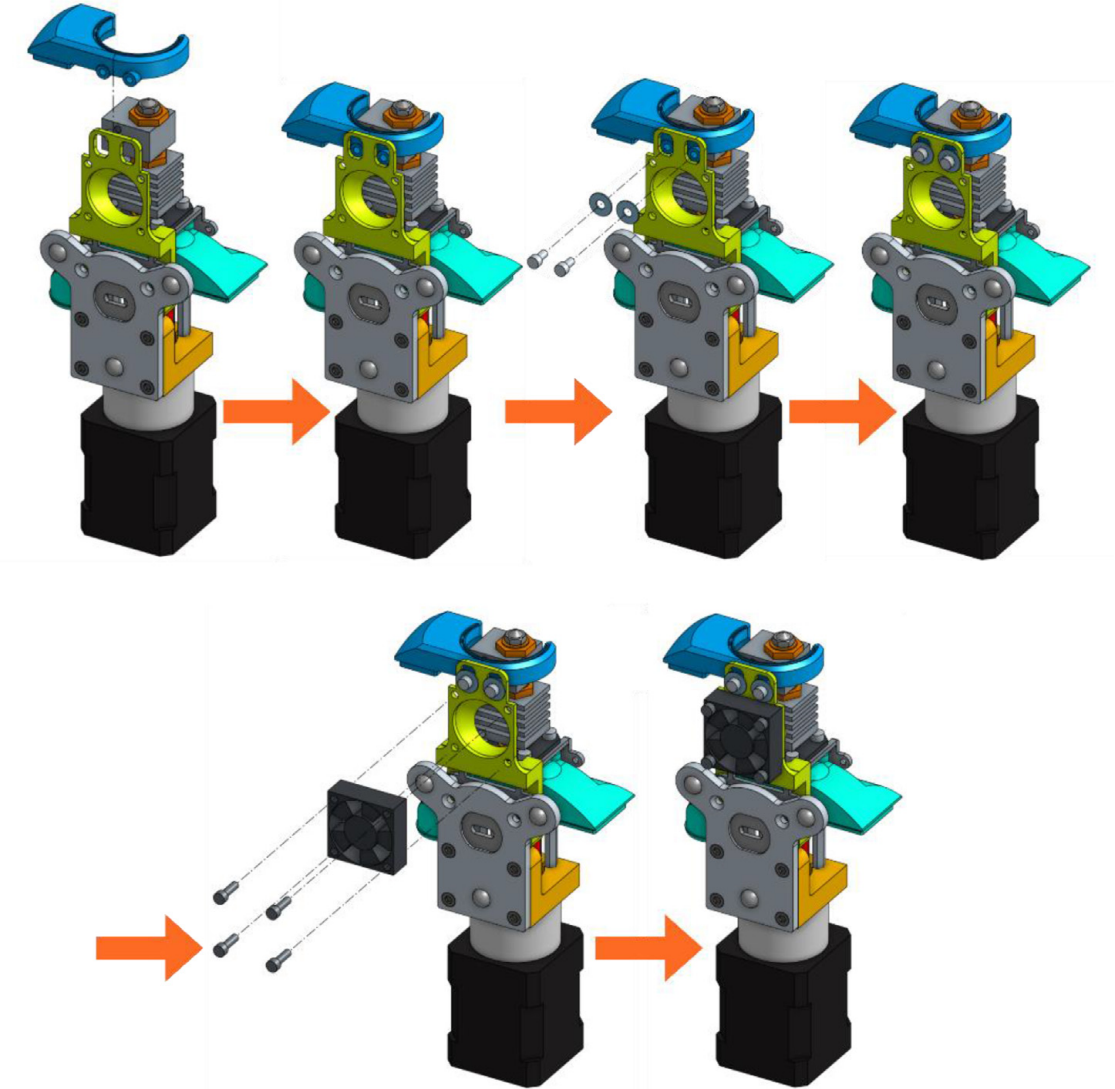
Adding the Cooling Pipe and the Heat-Sink fan.
• Finally, the Part-Cooling fan and the pellets fan can be placed and held in place with 20 mm long M3 screws. (Fig. 20 and Fig. 21). The funnel just needs to be inserted in the round end of the Pellet Case and can be further secured using a zip tie as it can be seen on Fig. 22.b.

**Fig. 20:**
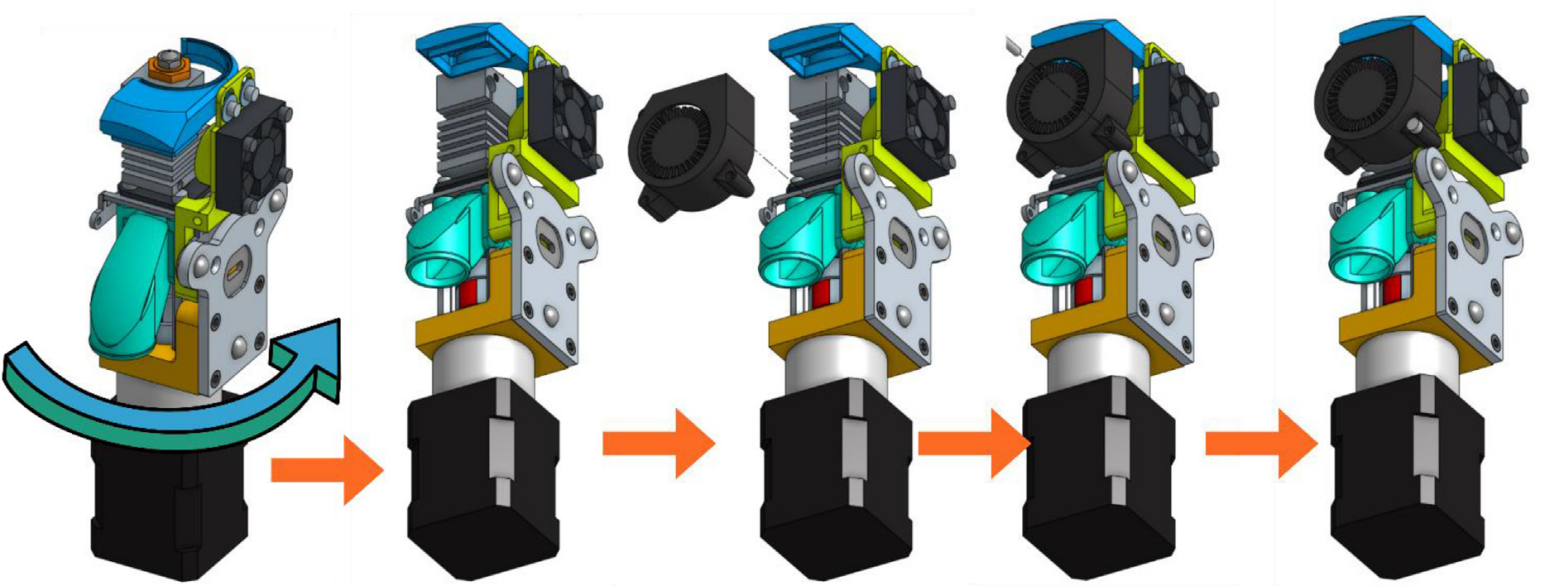
Mounting the Part-Cooling fan.

**Fig. 21:**
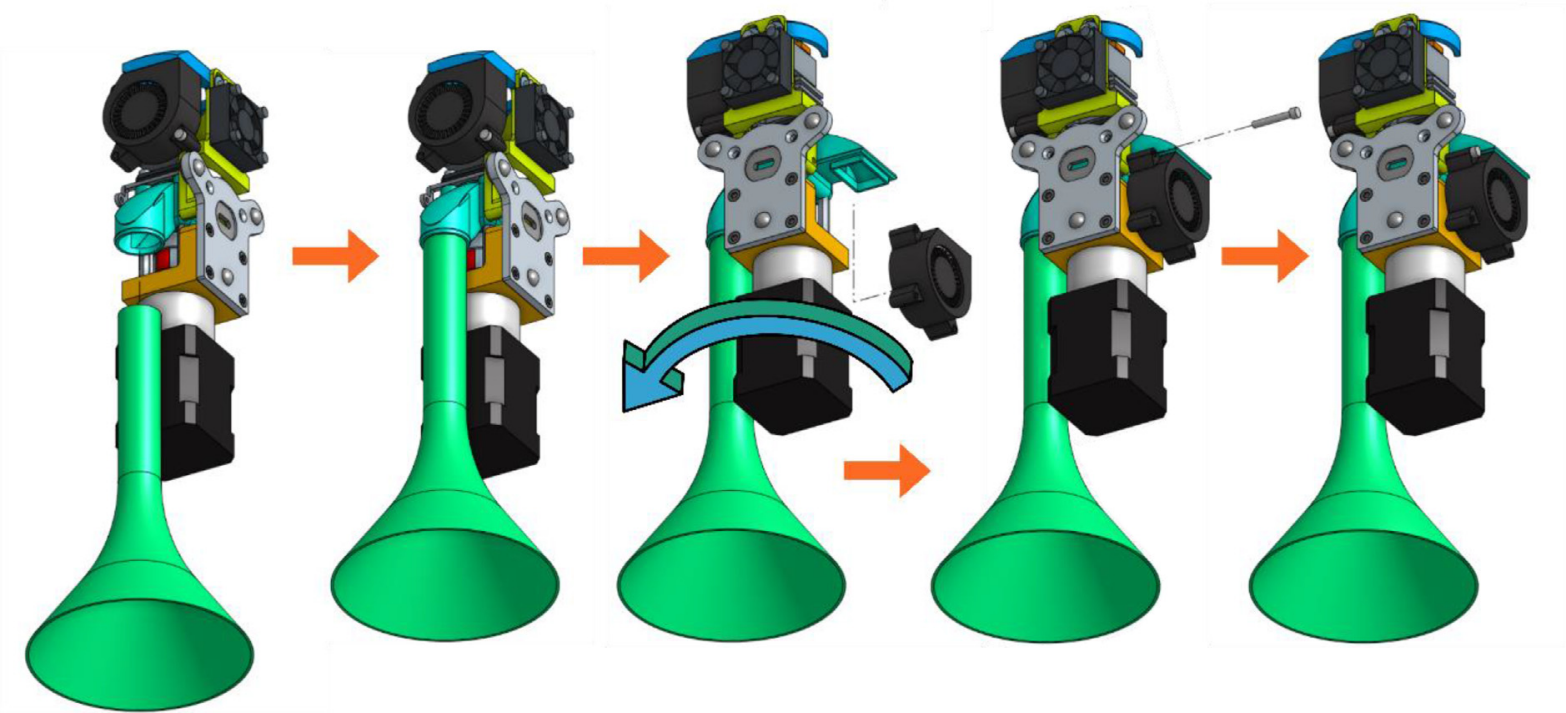
Addition of the funnel and Pellet Case fan.

**Fig. 22:**
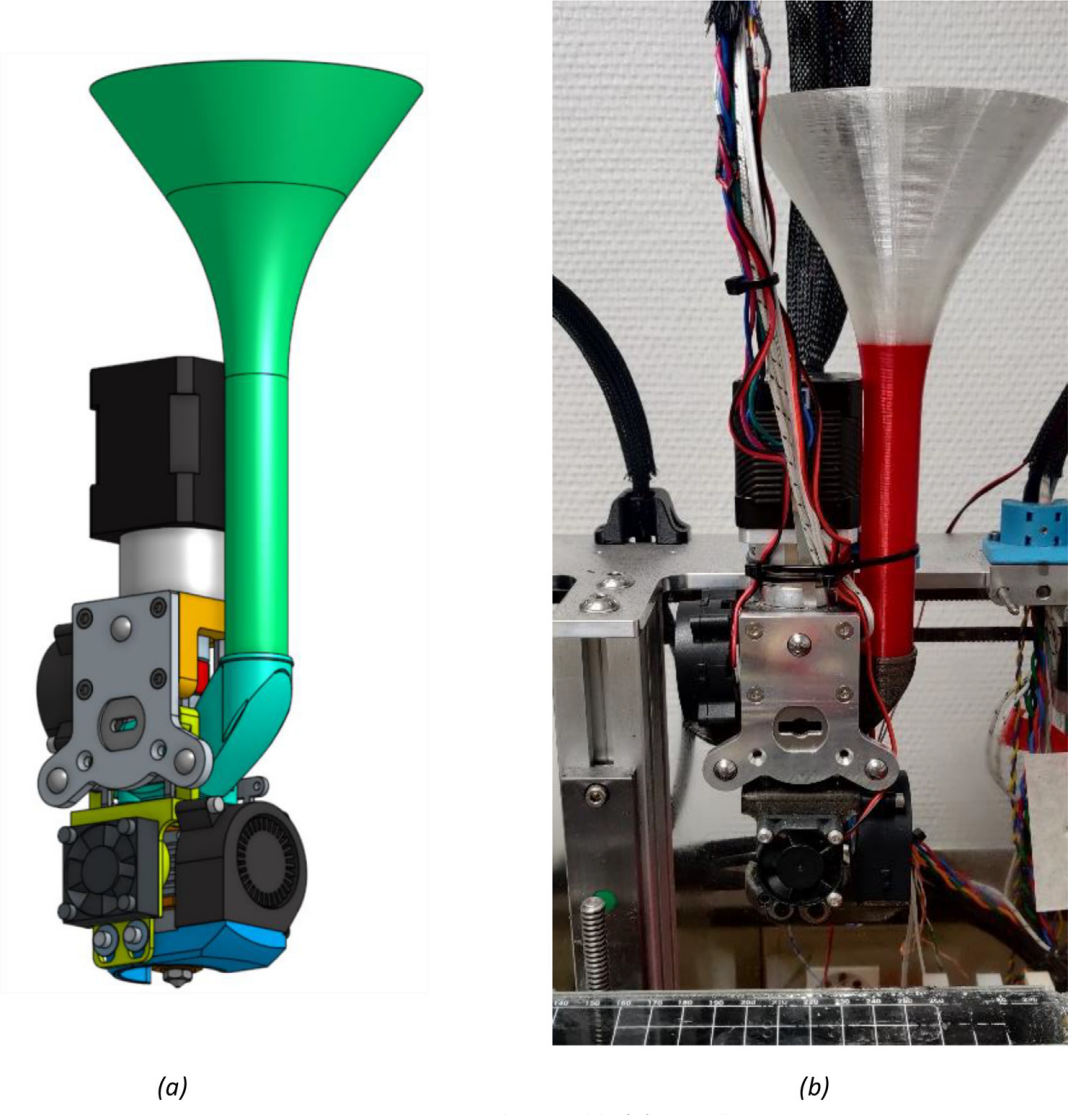
Final assembly (a); Final assembly with wiring (b).

### Wiring

**Fig. 23:**
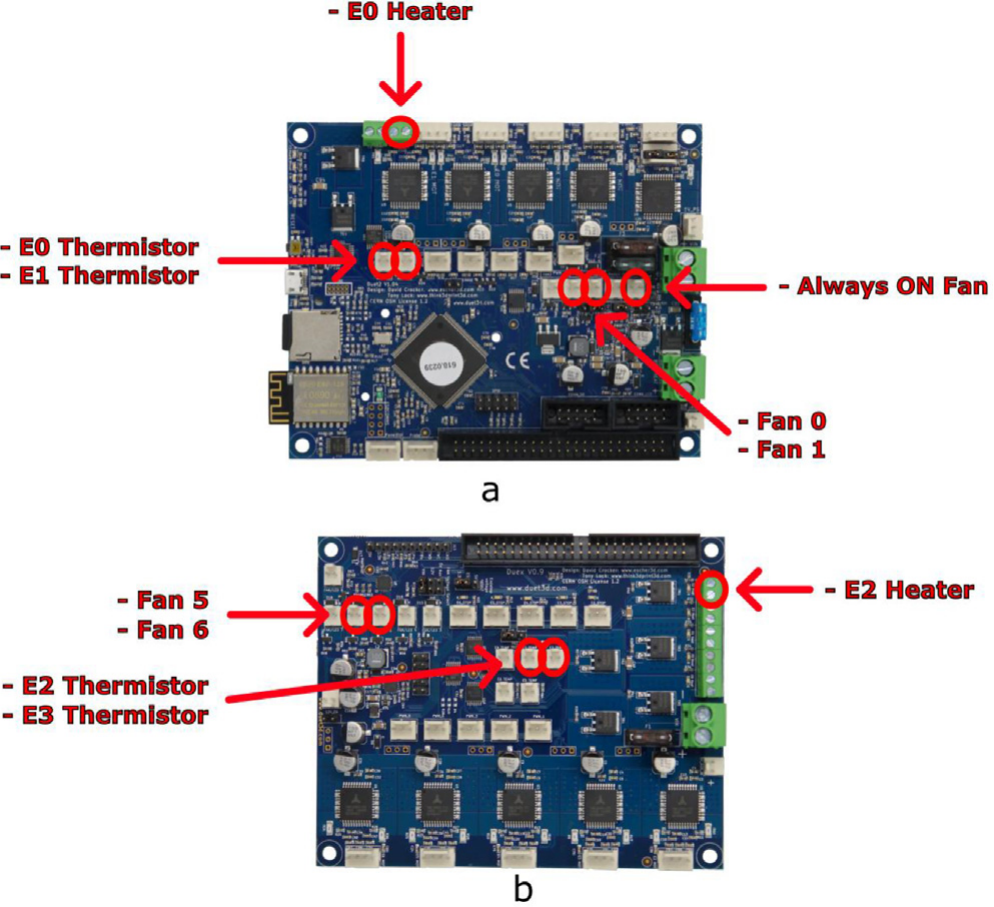
Duet 2 Wifi board (a) and Duex 5 expansion board (b).

In this example, there are six connection plugs reserved for the pellet extruder. Our tool requires a heating resistor, two thermistor temperature sensors and three fans. For this purpose, in addition to the Duet 2 Wifi (Fig. 23.a), we used the Duex5 V0.9 expansion board (Fig. 23.b), which normally allows the connection of supplementary heating elements and fans in order to use two additional extrusion heads on the Tool Changer. These are the two heads that we replace with one or two pellets extruders.

The pins used for both boards are given in Table 4 and are marked by red circles on Fig. 23.

**Table 4:** Pin list for connecting components on Duex 5 V0.9 and Duet 2 Wifi boards.

Board	Pin	Component
Duex 5 V0.9	E2 Heater	Heating resistor
	Fan 5	Heat-sink fan
	Fan 6	Part-cooling fan
	E2 Thermistor	Heating element temperature sensor
	E3 Thermistor	Heat break temperature sensor
Duet 2 Wifi	Always ON Fan	Pellet Case fan

It is also possible to use only the Duet 2 wifi card. In this case, the pins used are given in Table 5.

**Table 5:** List of pins for the connection of the components on the Duet 2 Wifi board only.

Carte	Broche	Composant
Duet 2 Wifi	E0 Heater	Heating resistor
	Fan 0	Heat-sink fan
	Fan 1	Part-cooling fan
	E0 Thermistor	Heating element temperature sensor
	E1 Thermistor	Heat break temperature sensor
	Always ON Fan	Pellet Case fan

**Fig. 24:**
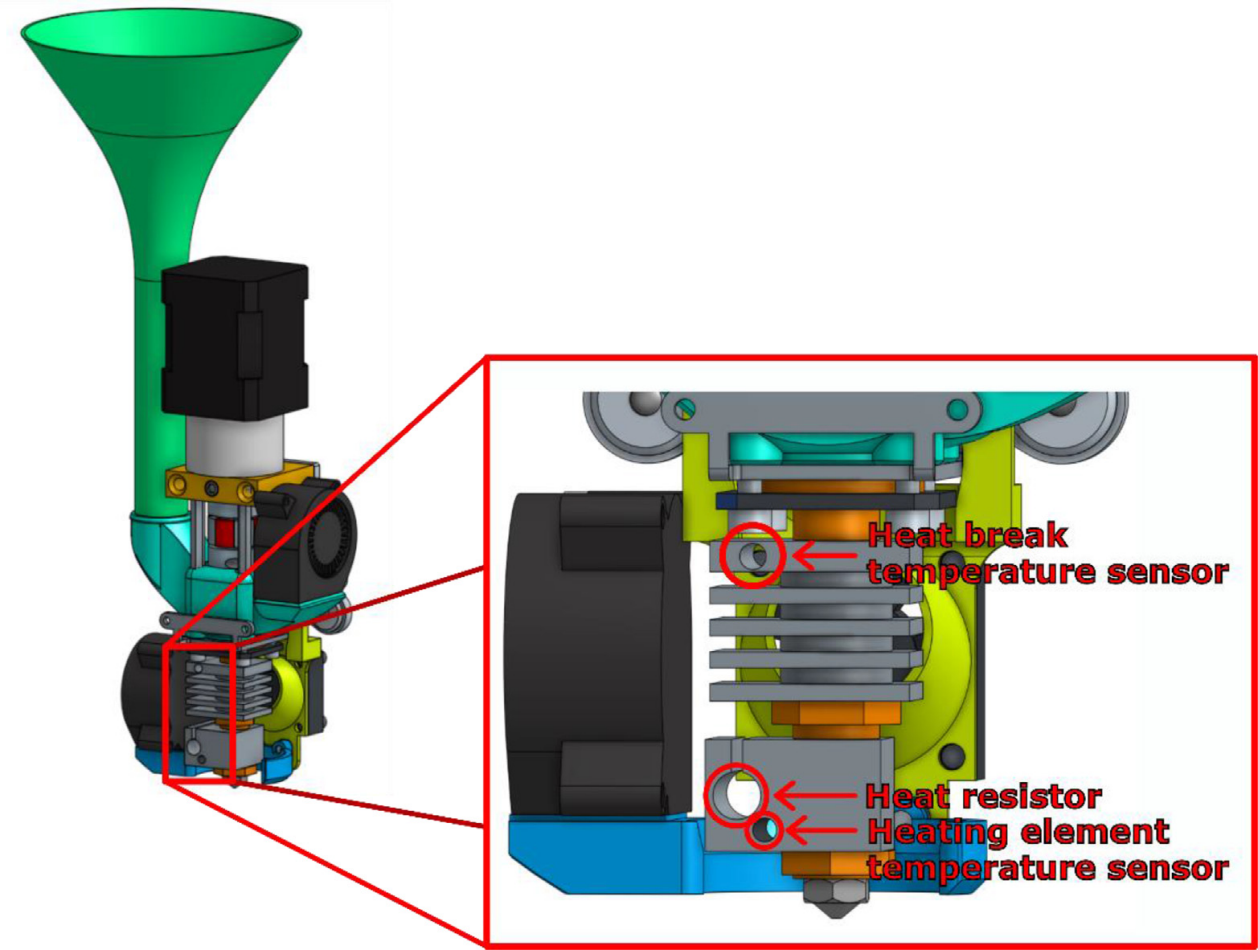
Insertion holes for heater and temperature sensor.

**Fig. 25:**
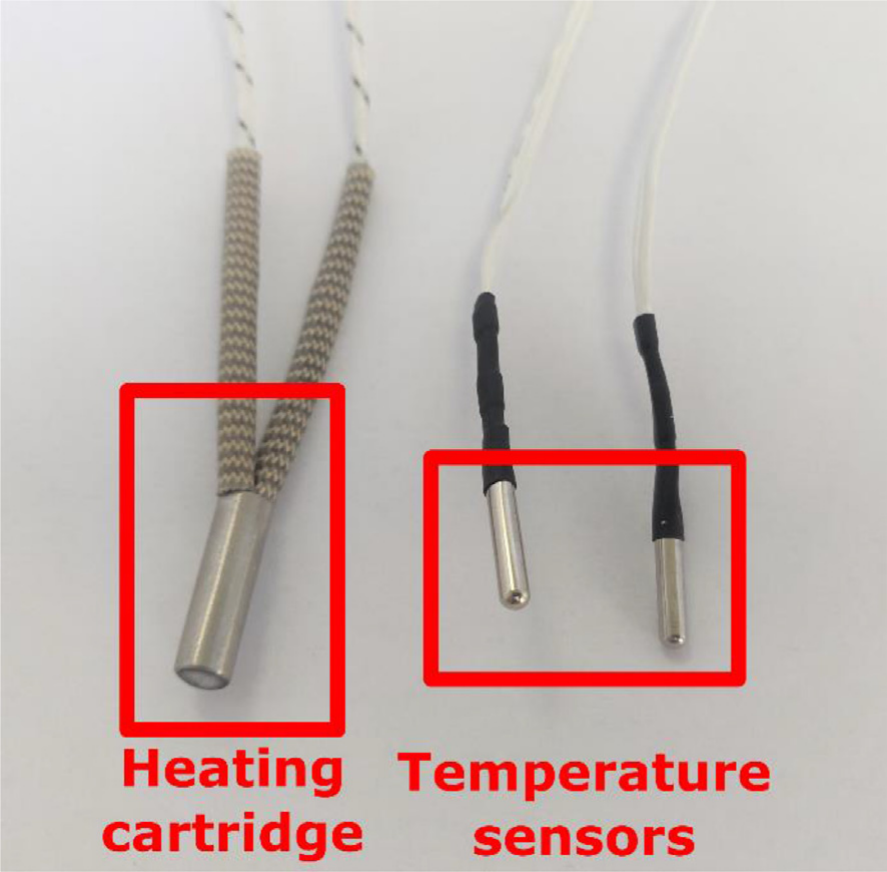
Heating cartridge and heat temperature sensors.

On the extruder’s heating body are three holes intended to receive the heat resistor, and the two thermistors as shown in Fig. 24. These elements are included with the Mahor Pellet Extrusion V3 (Fig. 25). These components might not have sufficiently long wires. These can be extended using some simple tin soldering.

### Extruder configuration

Once the assembly and wiring are done, modifications in the control of the board and tuning are needed. The necessary code and explanation are given below.

#### config.g file

The “config.g” file is the configuration file present in the memory of the Duet 2 Wifi board and that allows the setting of a very large part of the printer firmware. This file is entirely written in G-code and is read at the startup of the card. However, each setting can be modified on the fly by sending the same G-code instructions in the terminal provided for this purpose. This allows the user to modify a parameter while printing in order to make a fine adjustment, but also to save a whole profile of parameters for storage. In the following parts, the parameter lines that must be entered to use the pellet extruder are given.

An example of the complete “config.g” that can be used for the Duet 2 Wifi + Duex 5 configuration, with the modifications presented below, is included in the Zenodo file repository whose link is given at the beginning of this article.

##### Fan/temperature sensor association

The fan placed on the heat break must start automatically when the temperature of the top thermistor (the one inserted in the heat sink) exceeds 70 °C. The rotational speed of this fan will allow to adjust the temperature gradient along the extrusion screw.

• The G-code command “M106 P5 S255 H3 T70” is already present in the config.g file provided by E3D for the tool changer. Nevertheless, the parameter “H” must be modified to correspond to the temperature sensor of the thermal barrier (H1 on the Duet2, H4 on the Duex 5).
• The fan setpoint “S” varies between 0 and 255 and allows control of the air flow and indirectly of the temperature. This value must be adjusted empirically so that the temperature near the screw inlet is high enough to soften the pellets without however melting them and risking a plug. The setting depends on the type of polymer binder of which the granules are made, but also on the nature of the powder and the proportion in which it has been mixed. A temperature reading between 130 and 140° allowed us to run the print from pellets loaded with 316L stainless steel powder. The fan setpoint used for this was “S150″.

##### Adding a second temperature sensor

To allow the reading of the temperature upstream of the screw and thus help with the tuning of the set point described above, a secondary thermistor can be inserted in the hole at the top of the heat sink as shown in Fig. 24. To display it in the Duet Web Interface, it needs to be declared using the “M305” Gcode command as in this example: “M305 S“T2_cold” P105 X5 T100000 B4725 C7.06e-8”.

**Fig. 26:**
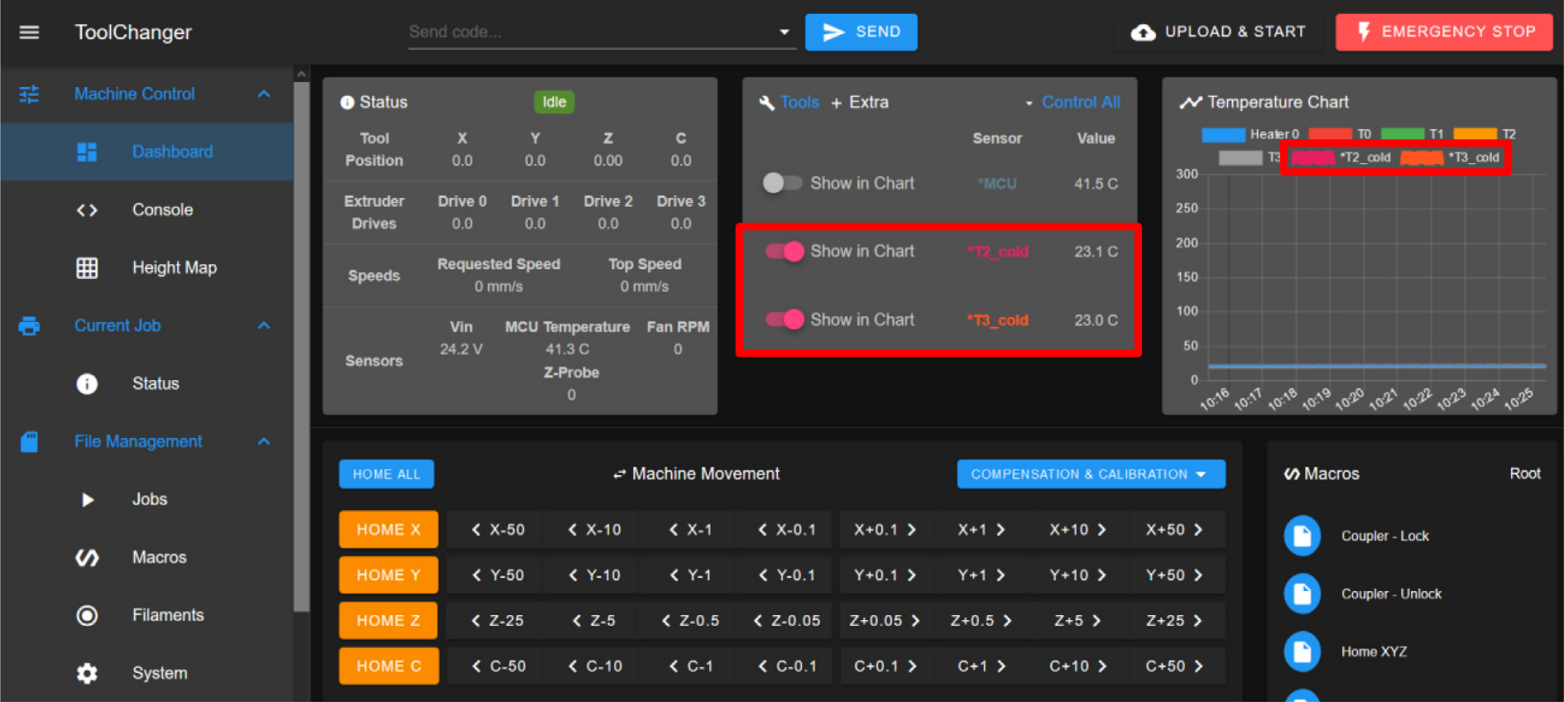
Addition of two temperature reading “T2_cold” and “T3_cold” in the Duet Web Interface.

Since it is not linked to any physical heater, the “P” parameter must be filled with a value higher than 100 to declare a virtual heater. The interface will feature two new sensors in the “Extra” tab and their value will be plotted along the others in the “Temperature Chart” (see Fig. 26).

##### Tool offset

In order to use the tool accurately, the offset between the position of the extrusion head and the position of the tool carrier must be taken into account. In the vertical direction Z this offset is particularly important for the success of the initial layer of the print. This is because the distance of the extrusion head from the heating plate determines the thickness of the first deposited filaments, which has a great influence on the pressure conditions at the outlet of the extruder and thus on the flow rate of the material. In the directions of the horizontal plane X and Y, the correct adjustment of the offsets is essential to make several tools work together on the same part. For example, to make multi-material prints.

An example of G-code to set this parameter is: “G10 P0 X-9 Y39 Z-5″, where the parameter P designates the tool in question and X, Y and Z are the offsets in the three spatial directions.

##### Method for measuring the “z offset”

**Fig. 27:**
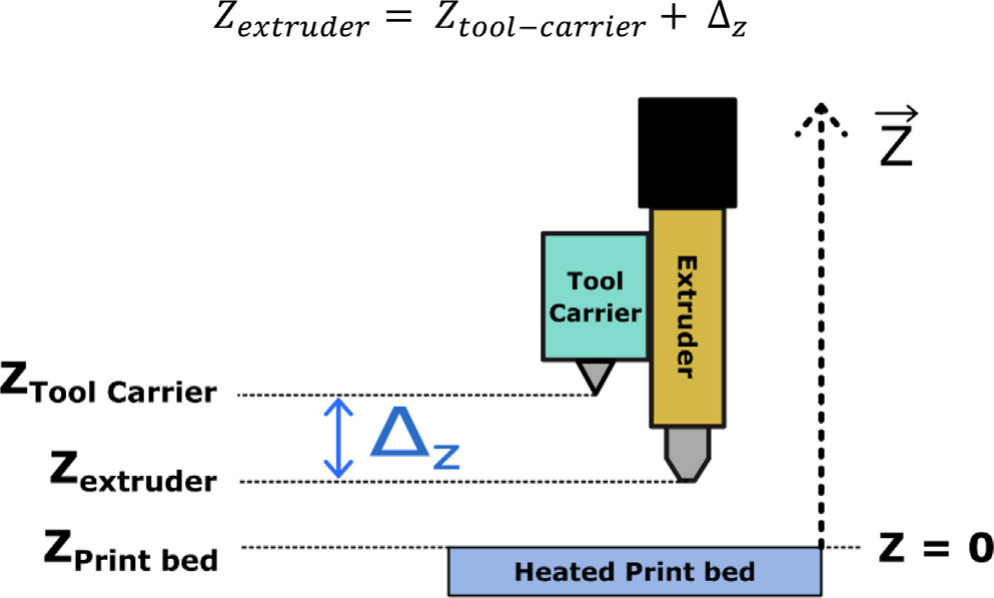
Offset between the nozzle tip and the tool holder in the vertical axis.

As shown in Fig. 27, the offset Δz is defined as follows:

$$Z_{\mathrm{extruder}}=Z_{\mathrm{tool}-carrier}+\Delta_{z}$$

$\Delta_{z}$ is therefore an always negative value.

The easiest method to measure this “offset” is to use the machine itself. This is done by first gripping the extruder with the tool carrier and slowly bringing the extrusion nozzle close to the glass plate until it makes contact (contact can be appreciated by sliding a sheet of paper between the nozzle and the plate until it is pinched). This movement must be done manually using the movement controls on the Dashboard in the “Duet Web Control” interface. It is important to note that it is the plate that is moved vertically because the tool can only travel horizontally. In any case, the relative position of one with respect to the other is modified.

Then we can read the $Z_{\mathrm{measured}}$ position displayed on the interface. The new offset value to enter is:

$$\Delta_{z}={\mathrm{ep}}_{\mathrm{paper}}+\Delta_{z,old}-Z_{\mathrm{measured}}$$

With ${\mathrm{ep}}_{\mathrm{paper}}$ the thickness of the sheet of paper used and $\Delta_{z,old}$ the value of the offset previously in memory. The value $Z_{\mathrm{mesur}\overset{´}{e}}$ displayed must then be equal to ${\mathrm{ep}}_{\mathrm{paper}}$.

##### Pressure advance setting

Pressure advance is a setting implemented in most 3D printing FFF firmware. It is meant to compensate for the elasticity of the filament as well as the mechanical play in the tube and in the stepper motor that, combined, creates a transient flow behavior. For classical printing polymer, it has an impact on the homogeneity of the line width. When starting a segment, the flow is delayed in comparison with the movement of the head and the deposited line is too thin. Similarly, the flow is stopped late and progressively at the end of the extrusion, resulting in lumps or stringing between parts.

In the case of pellet printing with an extrusion screw, similar behaviors are observed, especially with viscous polymer blends. However, the consequence for this kind of material is not necessarily inconsistent width. Rather, it impacts the cohesion of the newest line with the last ones and with the previous layer. It also tends to give porous or damaged structures. To solve this issue, the Pressure Advance setting can be used.

Pressure advance allows to impact the extrusion speed linearly in relation with the acceleration of the printing tool:

$$\mathrm{Spee}d_{\mathrm{effective}}=Speed_{\mathrm{intial}}+\left(k*acceleration\right)$$

This will increase the flow when speed is increasing, meaning that the extrusion could be lagging behind. Subsequently, the flow is decreased on deceleration.

On the RepRap firmware, the tuning of this value is done using the M572 G-code command but its value will heavily depend on the material used as binder. Effective parameterization procedures and extended information are available from the duet3D website.^17^

## Operation instructions

Printing from pellets raises several challenges that we propose to solve by effectively using the capabilities of the Tool Changer printer and its control board. One obstacle inherent to the method is the management of the extrusion rate, whose behavior depends non-linearly on many parameters [63]. In addition, the adhesion of the extruded filaments to each other is not as easily achieved as it would be with a conventional polymer feedstock, especially when printing heavily filled pellets with a powder from a different material.

Once adapted to the printer, the extrusion head can be fitted to the Tool Changer's tool holder, which will move it in the X and Y directions. The control of the whole system is similar to a classic Material Extrusion head. The extrusion command from the filament-pusher system (gear and roller) is simply applied to the stepper motor that is coupled to the extrusion screw and executes the instructions dictated by the G-code. To obtain a good extrusion, the Slicer software (Ultimaker Cura, Simplify 3D, Prusa Slicer, etc…) must be configured in such a way as to obtain a suitable ratio between the rotation speed of the extrusion motor and the movement speed of the nozzle in relation to the plate. This ratio is controlled by the “Flow” parameter in Cura or “Extrusion multiplier” in Simplify3D.

In this part the settings and macros that can be used to achieve a successful pellet print are presented.

### Grabbing and dropping the tool: “tpre.g”, “tpost.g” and “tfree.g” files

The size of the extruder has made it necessary to increase the size of the “TC-Dock_L” part. As a result, the distance that the head must travel to dock or release the head must be slightly shorter. In the system proposed by E3D for the Tool Changer printer, the capture and release of a head are carried out by three macros that we need to modify: tpre.g and tpost.g allow the object to be picked up, and tfree.g allows it to be released. These scripts contain the G-code instructions that govern the positioning and locking of the tool holder latch system.

In the tpre.g macro, the length of the approach path to the head is reduced. The travel speeds are reduced as well because the complete extruder weighs around 850 g making it heavier than the standard E3D tools. Finally, the downward movement of the plate is increased to avoid collision with it. In the tfree.g macro, only the approach movement and the movement speeds are modified.

Another important point is the addition of a passage through the center of the plate for the pause (“pause.g”) and cancel (“cancel.g”) macros. Indeed, the length of the extruder makes it likely to collide with the threaded rod that ensures the vertical displacement of the plate.

### Macros

Printing with filled polymer pellets confronted us with two main problems. The first is the blockage of the nozzle, which is pretty common with FFF printing. This was caused by a too coarse granulometry and can be solved by using larger sized nozzle. We also realized, during our experimentations, that too low a flow could lead to a clogging as well because of fine particles accumulating at the outlet of the heating body.

Another difficulty is linked to a well-known phenomenon in grain mechanics and powder flow. Inside a silo that ends with a hopper (the area where the cylinder narrows to a conical shape), an arch can form and support the granules on top. This is due to the fact that the stress distribution inside a powder or granular structure is not isotropic [64] and is directed to the cylinder wall. The force that acts on the pellets at the bottom of the silo is not the sum of the weight of all the granules above it as it was known for a long time already [65]. This results in a blocking of the pellets that depends on several parameters such as the cylinder diameter, the hopper slop or the presumed shape of the arch [66], [67]. A similar effect can happen in the funnel and the pellet case, which results in a discontinued flow of matter or a total lack of it.

The occurrence of this problem is not systematic and depends strongly on the granules, their shape and size. However, to increase the chances of a successful print run, feedstock feed flow can be improved by using macros that are inserted into the G-code and that trigger pauses in order to drop any stuck pellets from the funnel or to purge the nozzle before resuming printing.

To do this, a macro named “shake_and_purge.g” can be called at regular intervals throughout the printing process. It orders the head to move away from the part being printed and shakes it vigorously by making short round trips on the X axis. This has the effect of moving all the granules upstream of the screw and into the tank to unblock them. At the end of this movement, the tool exits the printing bed and purges a small amount to re-prime the head (i.e., to fill the nozzle and bring the material right to its exit). Finally, the nozzle can be cleaned by rubbing it against a brush or by moving it close to the glass plate. The printing then resumes normally and this sequence can be restarted later.

The use of vigorous shaking is made possible by the small size of the extruder. Other systems, such as the Pollen printer, which have more space, use a shaker driven by an independent motor. This solution seems difficult to adapt to our system and in such a small space, without disturbing the arrival of the granules.

### Calling macros according to the quantity of extruded material

The macro shake_and_purge.g is useful to prevent blocking of the pellets, which can happen regularly during the printing process. In this case, the shake must be performed several times. First of all, it is possible to use the post-process functions of the G-code offered in the Cura software. The script “insert at layer change” allows to insert one or several G-code instructions at each layer change. To call a macro, the G-code command to send is: “M98 P'shake_and_purge.g'”.

**Fig. 28:**
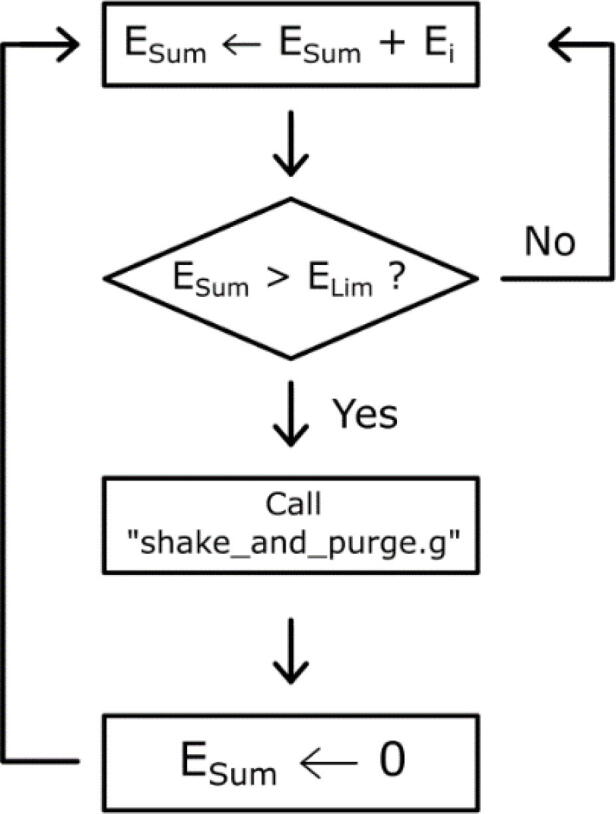
Logical diagram of the extrusion instruction count.

However, for surfaces that require several minutes of extrusion, it may be necessary to call this sequence more often. This can be done using a Python script that reprocesses the G-code file produced by Cura before sending it to the Tool Changer. Given a G-code file, this script counts the extrusion instructions and inserts the macro call whenever this sum exceeds a value chosen by the user (Fig. 28).

### Example of slicer setup with Ultimaker Cura

The preparation of the control of the extruder to carry out a printing from a 3D numeric object is done in a software called Slicer. Starting from a file that describes a geometrical object (.STL), this software will produce the sequence of instructions of displacement and extrusion, which will make it possible to recreate this object physically. The object is “sliced” into several layers that are themselves composed of multiple paths along which the filaments of material that make up the final part are deposited. This type of software offers a multitude of parameters that will regulate different aspects of the printing work and allow the success of the latter.

In this section several changes in the printing parameters are presented. These are specific to the use of the Mahor extrusion head with the Tool Changer. The Ultimaker Cura software is chosen for the example but the features used are present in most existing Slicer. A list of the main parameters that were modified to print the PolyMIM 316L pellets is shown in Table 6.

**Table 6:** List of parameters used for printing PolyMIM 316L granules.

Printing parameter for Cura: PolyMIM 316L, steel nozzle 0.6 mm			
Printing Temperature	Build plate Temperature	Printing Speed	Retraction Distance
210 °C – 220 °C	90 °C	20 mm/s	7 mm
Retraction speed	Nozzle size	Layer Height	Flow
30 mm/s	0.8 mm	0.3 mm	85%

#### Operating temperature

The elements that constitute the Mahor pellet extruder can easily operate up to a temperature of 250 °C, which makes it possible to work with a large part of the polymers used in 3D printing. However, the choice of extrusion temperature and heating plate depends greatly on the material used.

The temperature homogeneity during the printing process remains a crucial point. Indeed, the flow rate of the extruded material depends greatly on the pressure conditions at the nozzle outlet. These conditions may vary with the temperature of the enclosure, of the glass bed and of the part already printed.

#### Printing speed

One of the difficulties specific to extrusion with a screw is that the relationship between the rotation of the screw and the quantity extruded is not fixed. Indeed, the latter depends a lot on the rotation speed of the screw and for the same number of revolutions, the quantity of extruded material can vary with the speed. This effect limits the speed variations for the same print because the extrusion coefficient (rotation speed/travel speed) should be adapted for every time. To overcome this limitation, it is more practical to carry out all the printing movements at the same speed.

#### First layer and adhesion to the print bed

This is a decisive step for many printing processes. In our case, the E3D printer has a glass plate that needs to be heated to soften the part of the model in contact with it and improve its adhesion. Moreover, our starting material, PolyMIM 316L granules, has a very viscous and not very adhesive behavior. The deposited bead of material tears easily and tends to curl without adhering to the printing plate.

The adjustment of the extrusion coefficient is even more crucial for this first layer and must be done independently of the rest of the printing. It may be possible to slightly increase this coefficient to obtain a slight over-extrusion that will improve the adhesion. It is also advisable to create an additional skirt around the part (a “brim” in Cura) to increase the contact surface. Finally, the user can make use of adhesives (glue stick or 3D printing spray) applied to the plate before printing. This thin layer can also be dissolved with isopropyl alcohol to make it easier to remove the part and avoid forcing it, which could damage or deform it.

#### Inflated nozzle diameter

In a similar way to what is done for the adhesion of the first layer to the plate, the flow of material during the printing must be sufficiently high to allow the deposited thread to hold on the preceding layer. This is also required so that it does not tear and that it binds with its neighbors. Thus, we also increase the ratio of extrusion (the rotational speed of extrusion motor over the displacement speed of the head) to be in light over-extrusion. Consequently, the deposited wire diameter obtained is higher than the nozzle diameter, which must be considered in order to obtain a good deposition. The slicer software is therefore provided with a fictitious and slightly wider nozzle size that corresponds to the thickness of the deposited strand.

### Debinding and sintering

Once the parts are printed, the water debinding step must be conducted as recommended by the manufacturer PolyMIM. Specifically, a bath of a minimum duration of 10 h for 4 mm thick walls. In our case, 10 h and 24 h baths were performed for parts with a thickness ranging from 2 mm to 8 mm.

Then, the high temperature sintering process can be set up. For this work, two different environments were used for comparison. The first heat treatment was conducted under dihydrogen as recommended by the pellet’s manufacturer. This allows to protect the metallic material from oxidation. The second one was done in slightly degraded condition. Indeed, the environment used was composed of RH5 gas (10 % H_2_ – 90 % N_2_) and not pure H_2_. The main reason for this choice is to investigate the possibility of sintering with cheaper furnace solution. In effect, furnaces equipped to be used with an H_2_ environment are much more expensive than solutions for non-flammable gas such as RH5. This would greatly reduce the possibility of creating low cost parts.

**Fig. 29:**
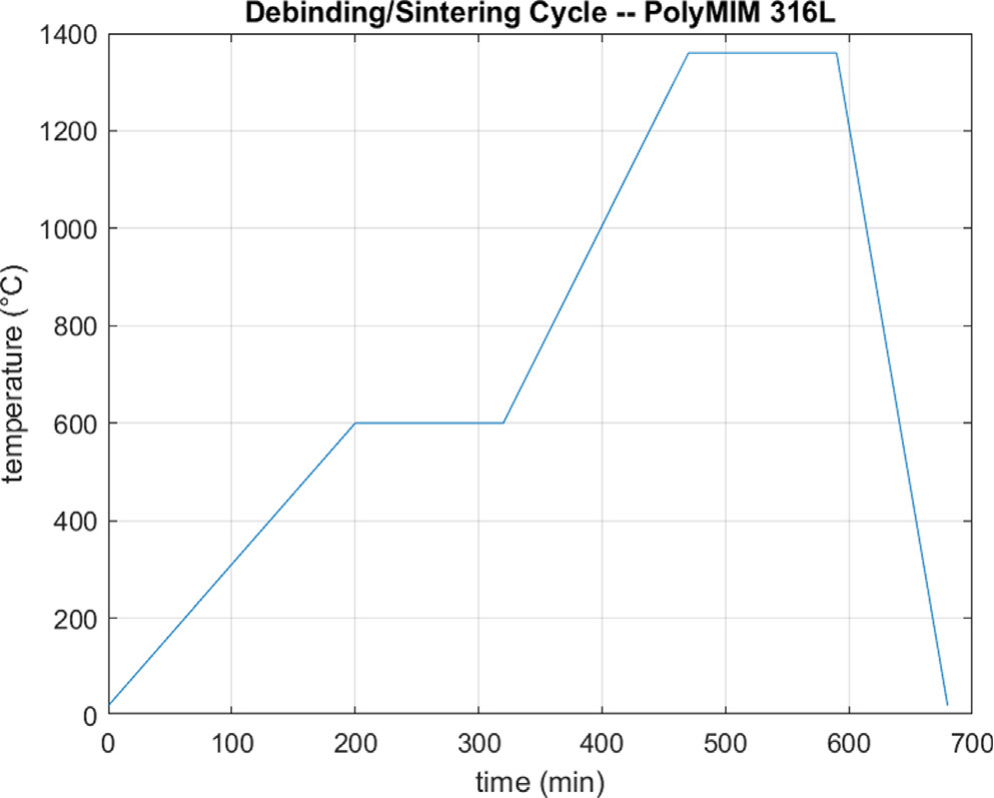
Debinding / Sintering cycle of PolyMIM 316L material.

In both cases, the densification of the parts was significant (above 93%). The test specimens showed retraction level ranging between 8% and 15% in the X and Y directions and a visual observation allowed to validate the protection against oxidation, particularly in comparison with samples sintered under air. Other parameters such as the temperature, heating ramps and holding steps remained the same (see Fig. 29).

### Safety precaution

The described process may present several risks for the user that are inherent to 3D printing activities and that must be acknowledged.

Firstly, burn injuries are a typical hazard to watch out for. Mainly, the hot end heating up to 250 °C must not be touched, but other parts such as the heat sink and the stepper motor can get very hot. For this reason, any maintenance task should be performed with a cooled down extruder. However, when clogged, the disassembly may require to melt the pellets and to manipulate a hot tool. In this case, heat insulating gloves must be worn.

Another hazard is pinching. Indeed, the E3D Tool Changer is capable of moving quite fast. No hands should be touching the printing area or the tools when a job is running.

Finally, the build instructions presented above require some electrical wiring. The printer must be shut off or disconnected from any power outlet when carrying these steps to avoid any electrical hazards.

## Validation and characterization

To illustrate the capabilities of the system presented above, various parts were printed with 316L stainless steel powder filled polymer pellets produced by the company PolyMIM. The fabrication and assembly of the parts, as well as the application of the process described in the first sections of this article, allowed us to successfully produce several samples. These samples served first for the adjustment of the printing stage and then, once the heat treatments were performed, for the mechanical characterization in traction of the sintered material.

### Green parts printing

**Fig. 30:**
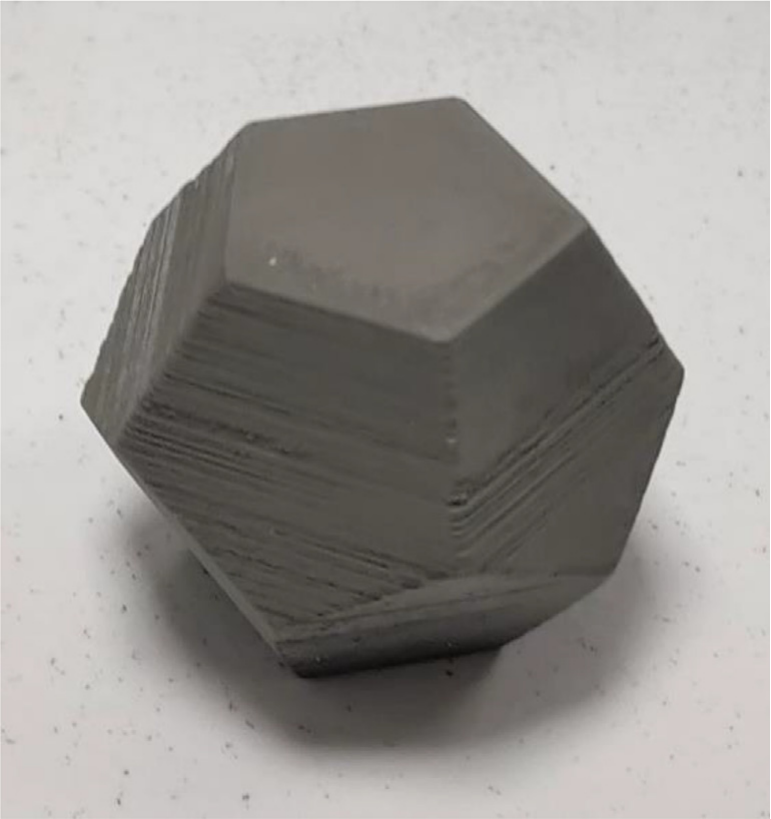
Calibration parts.

Examples of calibration parts printed to set the parameters of the printing job are shown in Fig. 30.

**Fig. 31:**
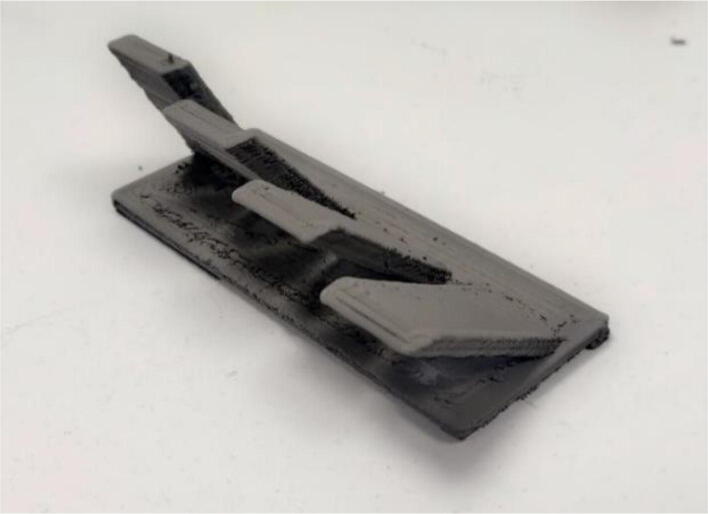
Test part of the printable inclination without support (30°, 40°, 50°, 60°) (green part).

To investigate the ability of the system to realize complex shapes, it was also undertaken to build inclined walls with different angles without using support structures. A maximum angle of 60° from the vertical was achieved. Similarly, test parts that concentrate all the difficulties encountered in additive manufacturing could also be realized (see Fig. 31).

**Fig. 32:**
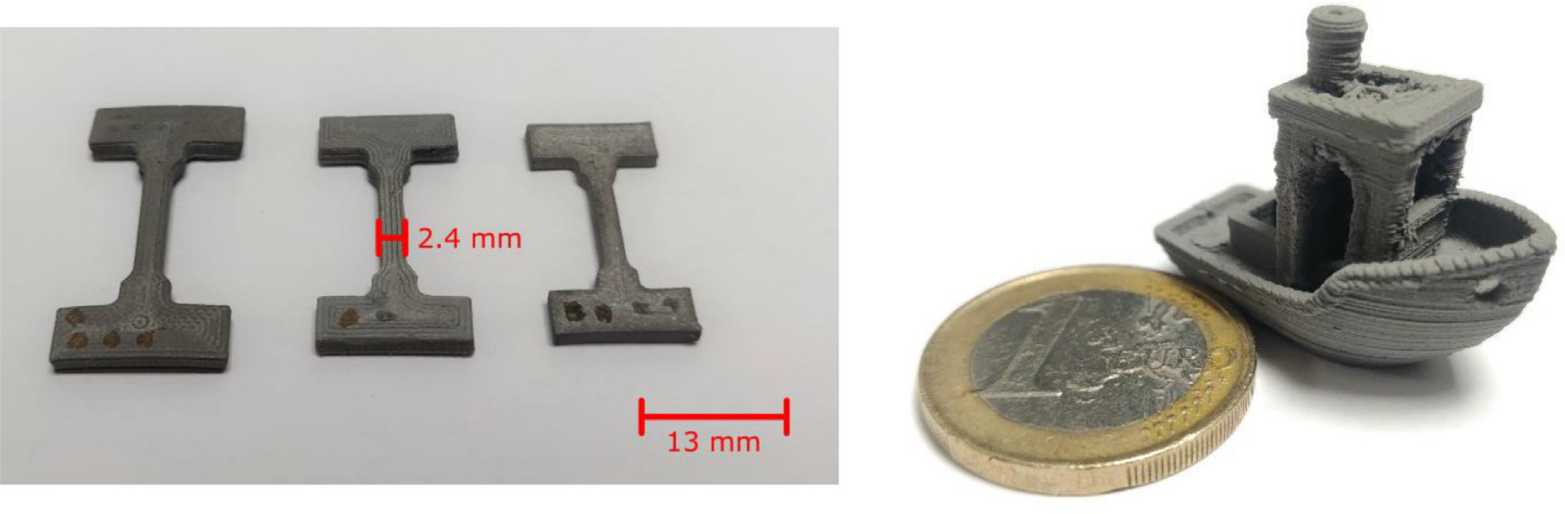
Benchy test print (green piece) and tensile specimens (sintered) for micro-tomography printed with a 0.25 mm diameter nozzle. (For interpretation of the references to colour in this figure legend, the reader is referred to the web version of this article.)

Different sizes of nozzles have been tested, with a maximum fineness of 0.25 mm diameter allowing the printing of small parts such as the tensile specimens shown in Fig. 32.a and whose working part has a thickness of 2.2 mm. Another example of the details achievable with the proposed machine is a model called Benchy (visible in Fig. 32.b), which has been printed to obtain the following dimensions: 26*14*18 mm.

Finally, the debinding and sintering steps, which allow to obtain a dense part made of the metallic material only, have also been conducted. The final density of the parts produced was measured on several one of them by using Archimedes buoyancy principle. It lies between 93 and 96% of the density of the reference 316L stainless steel [68], which is comparable to other work that uses a final thermal sintering step [69].

### Multi-material printing

The polymer binders used for our printing have a limited capacity to produce overhanging structures also called bridging. Also, the sintering step can cause significant deformation of the bridging structures. To remedy these problems, the printing of supports in a different material is a classic solution in Material Extrusion additive manufacturing. In order to produce a support that is useful for both printing and sintering, a feedstock of pellets filled with alumina powders can be used. Indeed, the ceramic material shows a higher melting point than most metallic materials and can be printed in a similar way.

**Fig. 33:**
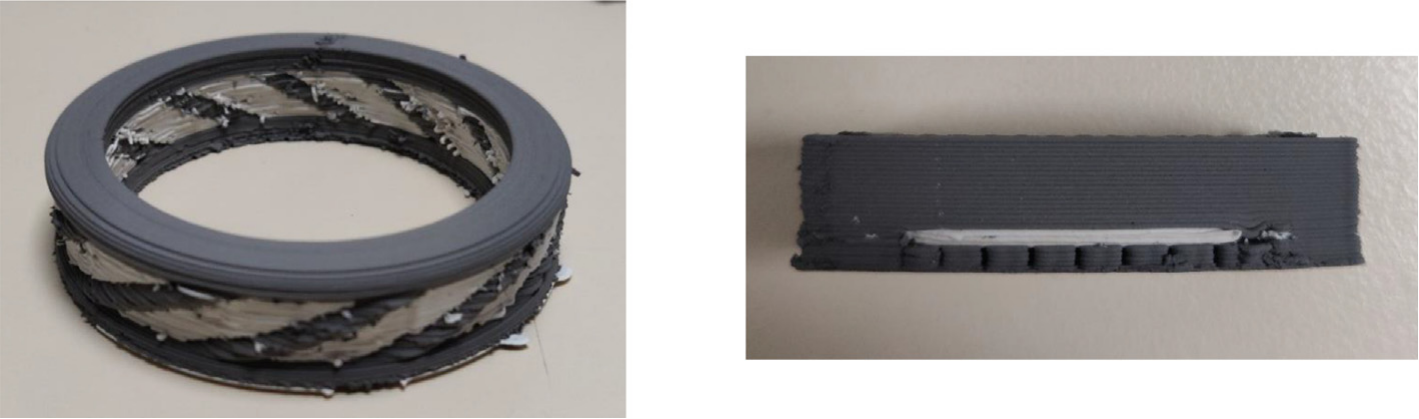
316L parts printed with alumina-filled polymer support.

To demonstrate the feasibility of the shaping part, we assembled and parameterized a second granulate extrusion head similar to the first one. The printing of parts in PolyMIM 316L with alumina support was then carried out to obtain more complex geometries that include overhangs as shown in Fig. 33. As stated before, the fine tuning of the X and Y offset was necessary to make it work properly. Similarly, macros that perform single purge and priming of the nozzle have been used to improve the quality of the print when switching from an extrusion to the other.

### Tensile mechanical characterization

**Fig. 34:**
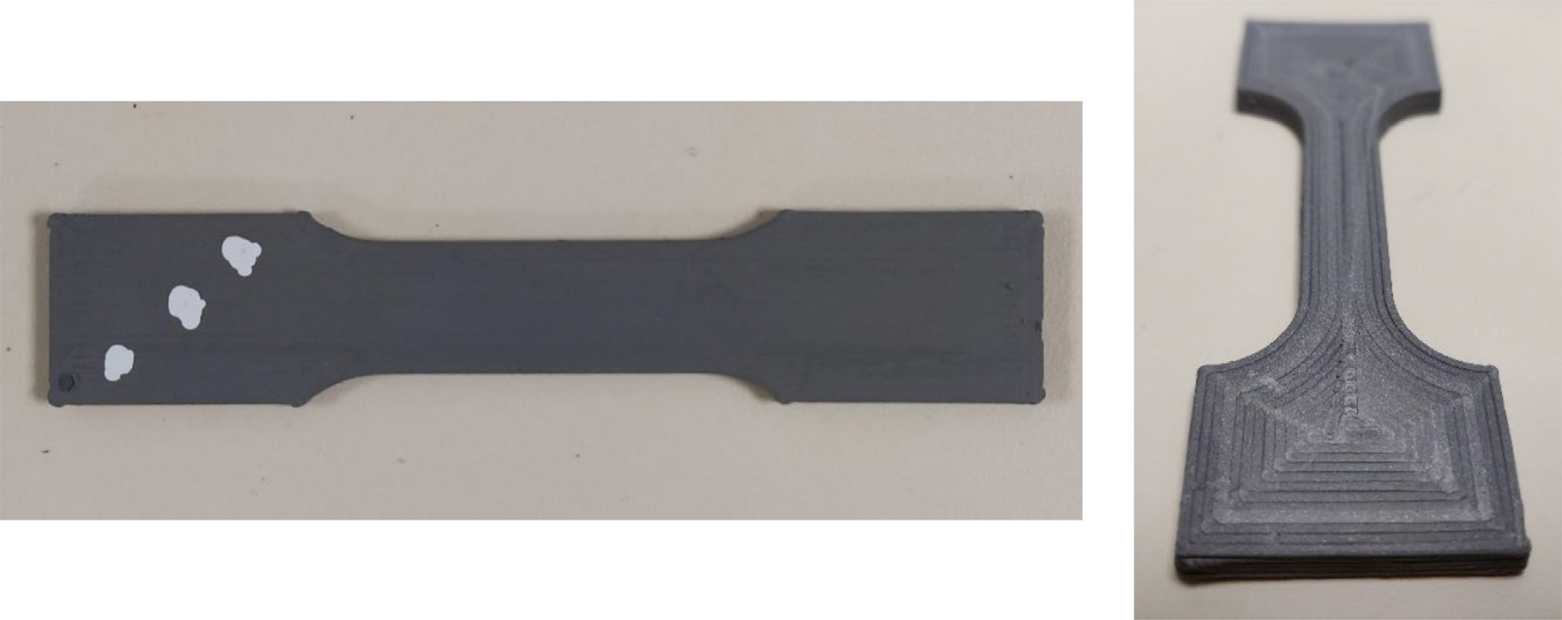
Tensile test piece. Green part on the left and sintered part on the right.

**Fig. 35:**
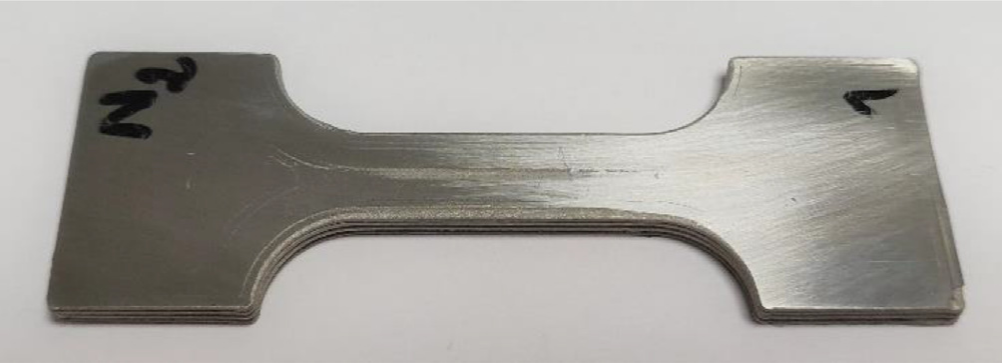
Sintered and polished tensile specimen.

To characterize the mechanical performance of a component obtained through the complete process, tensile mechanical tests were performed. For this purpose, specimens were printed from PolyMIM 316L pellets (Fig. 34 and Fig. 35), then the debinding and sintering post-processing steps were implemented as described previously with both RH5 and H2 environment. The reduced section of the green specimens is 30 mm long, 10 mm wide and has a thickness of 3 mm. The sintered parts were polished to obtain a perfectly flat cross-section and not to be deformed in bending during the test. A tensile test was then performed on an 50kN Instron bench equipped with an extensometer.

**Fig. 36:**
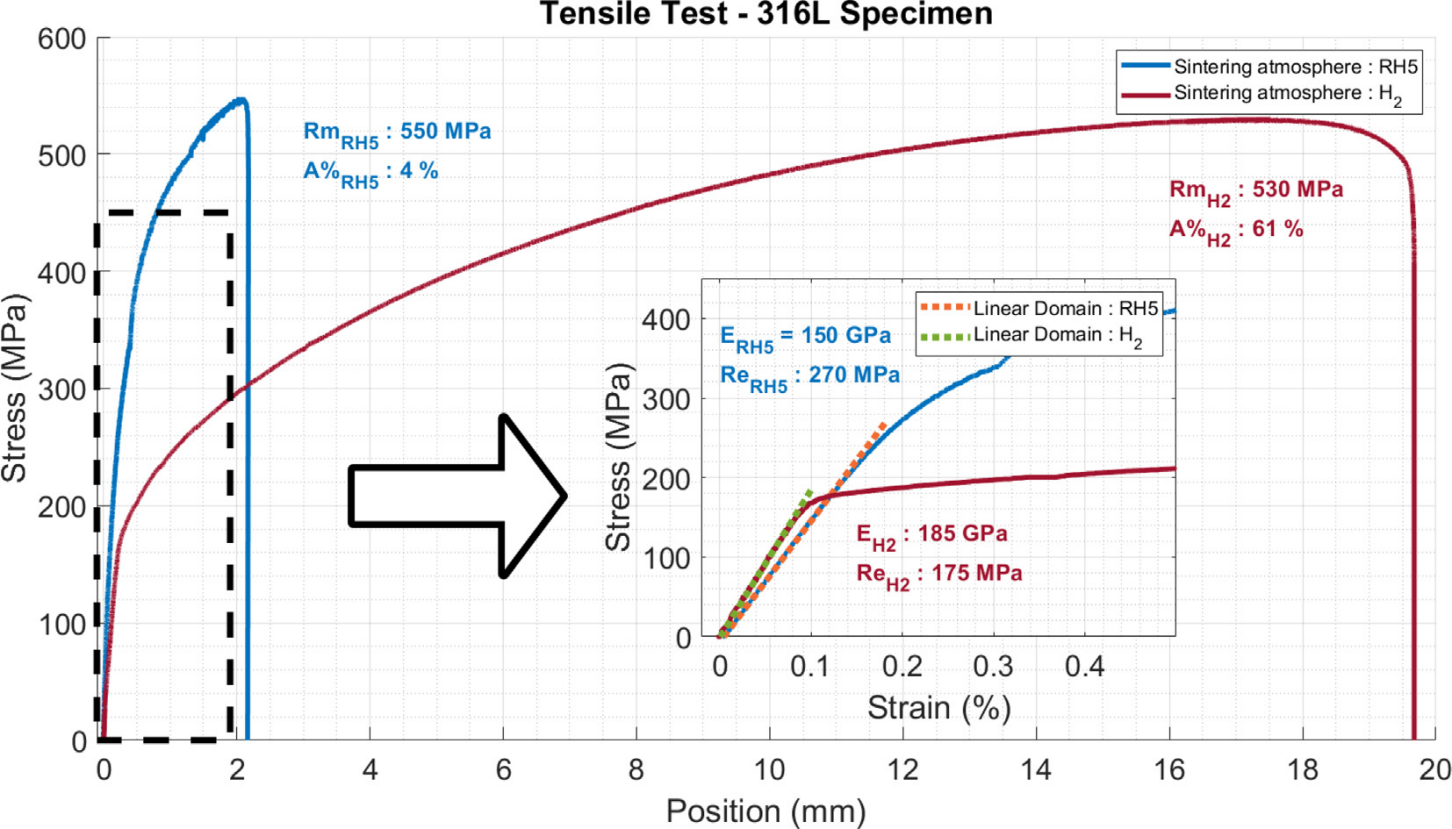
Tensile test on sintered specimen.

The graph in Fig. 36 shows the stress versus relative elongation and the test results are given in Table 7. Different printing strategy were used in order to investigate the impact of the orientation of lines and their arrangement in different patterns. For instance, by depositing the printing lines parallel or perpendicular to the direction of the force. These results will be presented in further work.

**Table 7:** Tensile mechanical performances of printed 316L steel specimen and comparison with the literature.

	Process	Ultimate Tensile Strength (MPa)	Yield Strength (MPa)	Young modulus (GPa)	Elongation (%)
This work	Granulate printing + RH5 Sintering	550	260	150	4
	Granulate printing + H2 Sintering	520 ± 25	130 ± 30	180 ± 10	46 ± 1
Gong et. al. [68]	FFF	465	167	152	31
Sadaf et. al. [69]	FFF	521 ± 16	252 ± 7	198	n. a.
Kim [70]	SLS	630	430	80^18^	31
Bartolomeu et. al. [71]	Hot Press	570	290	n. a.	34
Wu et. al. [72]	WAAM	583	339	n. a.	n. a.
Wang et. al. [73]	WAAM	550	418	n. a.	n. a.

18 It is important to note that this value was calculated using a graphical point collection from the figures provided by the authors, and may therefore be subject to measurement inaccuracies.

Fig. 36 presents the result of two specimens printed longitudinally (represented by the blue curve for RH5 and in red for H2), meaning that every line in the working zone are oriented parallel to the traction direction. The deposited lines can be observed on the green part on the right of Fig. 34.

Mechanical tensile tests allow a first comparison with stainless steel materials produced by other additive manufacturing technologies or with conventional methods (casting or deformation processes). The main quantity that allows a fair comparison is the Young modulus. In effect, its value will only depend on the porosity level of the final product whereas the yield strength and the ultimate tensile strength depend greatly on the processes specificities and its parameters such as the rate of work hardening or cooling speed, as well as on possible post processing treatment that can be done after additive manufacturing steps for example. In our case, the samples were cooled slowly by natural convection in the furnace environment, after the heat treatment. For this reason, we should only compare these results with materials in the literature that are densified using a sintering process.

For instance, the measured tensile strength for both H2 and RH5 sintering is found in the range of other fabrication techniques that involve sintering such as Metal Extrusion metal printing [68], [69], SLS [70] or Hot pressed samples [71] (see Table 7). Regarding the yield strength, reported values are 167 MPa, 252 MPa, 430 MPa, 290 MPa. We can see than even though the four articles used similar sintering processes (especially [68], [69]), large differences are to be noted between the different results. Ours values for the RH5 sintering are the closest with the yield strengths obtained by SLS and greater than other deposition process followed by debinding and sintering. However, the specimens sintered with H2 show lower values. When compared with values from WAAM process for 316L, a process that involves the fusion of the material, the tensile strength is found in close range with the given values (583 MPa and 550 MPa) but the yield strength is again much lower than what is find in the literature (339 MPa and 418 MPa). These differences call for further investigation.

Finally, the Young's modulus observed for the RH5 sintering remains below the expected values that can be found in the references previously quoted. The same observation can be made for the H2 treatment even though this gives better results. This probably indicates the presence of porosities that may be due to imperfect deposition during printing or to incomplete sintering.

It’s important to keep in mind that the sintering environment plays an important role in the densification and in the obtained performances. This is illustrated by the difference in young modulus and in maximum elongation between both gases. Dihydrogen sintering, which is expected to give better sintering results, greatly enhances these values. Results obtained during our test gave about 4% of deformation for RH5 and above 45% for H2, where references [68], [70] report above 30%.

These results were obtained on few specimens and need to be further developed. Extended characterization and improvement of the heat treatments will be conducted in future works in order to improve these last points. Nevertheless, the results presented here demonstrate the successful densification of the printed object as well as the removal of any polymer material to obtain a 316L stainless steel part that exhibits characteristic performances of such a material. The performance obtained with the RH5 environment are also satisfactory for the objective of reducing the cost of manufacturing.

## Conclusion and perspectives

This paper proposes a new low-cost solution for 3D printing parts from a feedstock in granular form. All the necessary components, the adaptation parts to be made and the software control using the E3D “Tool Changer” platform are presented. The first manufactured parts, as well as the results of mechanical characterizations carried out on the sintered 316L steel parts allow us to validate the quality of the filling and the capacities of the set-up.

Different challenges have been met such has the control of temperature gradient and the blocking of the granules. The difficulty of printing complex geometries from pellet has been tackled thanks to the use of extrusion control and multi-material printing of support.

The use of filled pellets with a similar binder polymer matrix allows the process to be extended to a wide range of materials. In principle, it is sufficient to mix the good polymer matrix with a powder of the target material. In particular, the companies PolyMIM and BASF already offer feedstock for MIM applications for various metal alloys and ceramic materials. These could be used even if there were not meant for 3D printing in the first place.

The use of the E3D Tool Changer gantry also makes it possible to consider the complexification of the process. Either by including different materials also printed from granules by adding a second Mahor extrusion head, or by adapting other types of manufacturing tools by taking advantage of the hardware and software flexibility of the Duet 3D board. In particular, the joint implementation of printing and CNC machining processes can greatly improve the dimensional accuracy of the resulting parts and bring additive manufacturing closer to industrial production standards.

## Declaration of Competing Interest

The authors declare that they have no known competing financial interests or personal relationships that could have appeared to influence the work reported in this paper.

## Footnotes

1 “Zetamix – Easy made ceramic and metal 3D printing,” *Zetamix*. https://zetamix.fr/en/ (accessed Sep. 27, 2021).

2 “Pollen AM | Pellet Additive Manufaturing” https://www.pollen.am/ (accessed Mar. 15, 2021).

3 “Cheaper 3D Printing with Pellets” All3DP Pro, Dec. 06, 2021. https://all3dp.com/1/cheaper-3d-printing-with-pellets/ (accessed Jan. 22, 2022).

4 “Atlas-H” Titan Robotics. https://titan3drobotics.com/atlas-h/ (accessed Jan. 20, 2022).

5 “DXR Series” https://www.hansweber.de/en/additive-manufacturing-systems/dxr-robotic-manufacturing-cell/ (accessed Jan. 20, 2022).

6 “Professional 3d printer | Delta WASP 3MT INDUSTRIAL 4.0.” https://www.3dwasp.com/en/professional-3d-printer-delta-wasp-3mt-industrial/ (accessed Jan. 20, 2022).

7 “Tumaker BIGFoot Pro Pellets - Impression 3D XXL” IT3D Group. https://it3d.com/fr/imprimantes-3d/grand-format/tumaker-bigfoot-pro-pellets/ (accessed Jan. 20, 2022).

8 “Direct3D – Pellet Extrusion” https://www.direct3d.it/ (accessed Jan. 5, 2022).

9 “Pulsar™ Pellet Extruder: Industrial High Flow Extruder” DYZE DESIGN. https://dyzedesign.com/pulsar-pellet-extruder/ (accessed Jan. 20, 2022).

10 “Bravo.,” Polylab. https://polylab.org/shop (accessed Jan. 20, 2022).

11 “The LILY Kit (Full DIY Set),” recycl3dprint. https://shop.recycl3dprint.com/products/the-lily-kit-diy-assembly-complete (accessed Jan. 20, 2022).

12 “Gigabot X – re:3D | Life-Sized Affordable 3D Printing” https://re3d.org/portfolio/gigabot-x/ (accessed Jan. 20, 2022).

13 “Duet3D.” https://www.duet3d.com/ (accessed Aug. 19, 2021).

14 “RepRapFirmware.org.” https://www.reprapfirmware.org/ (accessed Sep. 16, 2021).

15 https://e3d-online.zendesk.com/hc/en-us/articles/4402640504081-ToolChanger-Motion-System-Assembly-Guides.

16 https://e3d-online.zendesk.com/hc/en-us/articles/4406215782289-ToolChanger-Motion-System-Assembly-Videos.

17 https://duet3d.dozuki.com/Wiki/Pressure_advance.
